# Computational design of potent and selective binders of BAK and BAX

**DOI:** 10.1126/sciadv.adt4170

**Published:** 2025-09-05

**Authors:** Stephanie Berger, Erinna F. Lee, Tiffany J. Harris, Sharon Tran, Asim K. Bera, Lauren Arguinchona, Alex Kang, Banumathi Sankaran, Sila Kasapgil, Michelle S. Miller, Sean Smyth, Mariam Lutfi, Rachel T. Uren, Ruth M. Kluck, Peter M. Colman, Walter D. Fairlie, Peter E. Czabotar, David Baker, Richard W. Birkinshaw

**Affiliations:** ^1^ Department of Biochemistry, University of Washington, Seattle, WA 98195, USA.; ^2^ Department of Bioengineering, University of Washington, Seattle, WA 98195, USA.; ^3^ Institute for Protein Design, University of Washington, Seattle, WA 98195, USA.; ^4^ Department of Biochemistry and Chemistry, La Trobe Institute for Molecular Sciences, La Trobe University, Melbourne, VIC 3086, Australia.; ^5^ Olivia Newton-John Cancer Research Institute, Heidelberg, VIC 3084, Australia.; ^6^ School of Cancer Medicine, La Trobe University, Melbourne, VIC 3086, Australia.; ^7^ Molecular Biophysics and Integrated Bioimaging, Berkeley Center for Structural Biology, Lawrence Berkeley National Laboratory, Berkeley, CA 94720, USA.; ^8^ Walter and Eliza Hall Institute of Medical Research, 1G Royal Parade, Parkville, VIC 3052, Australia.; ^9^ Department of Medical Biology, The University of Melbourne, Melbourne, VIC 3052, Australia.; ^10^ Howard Hughes Medical Institute, University of Washington, Seattle, WA 98195, USA.

## Abstract

Potent and selective binders of the key proapoptotic proteins BAK and BAX have not been described. We use computational protein design to generate high affinity binders of BAK and BAX with greater than 100-fold specificity for their target. Both binders activate their targets when at low concentration, driving pore formation, but inhibit membrane permeabilization when in excess. Crystallography shows that the BAK binder induces BAK unfolding, exposing the α6 helix and BH3 domain. Together, these data suggest that upon binding, BAK or BAX unfold; at high binder concentrations, self-association of the partially folded BAK or BAX proteins is blocked and the membrane remains intact, whereas at low concentrations, dimers form, and the membrane ruptures. Our designed binders modulate apoptosis via direct, specific interactions with BAK and BAX and reveal that for therapeutic strategies targeting BAK and BAX, inhibition requires saturating binder concentrations at the site of action.

## Teaser

Selectively targeting BAK or BAX with potent designed proteins gives differential functional outcomes that inform therapy design.

## INTRODUCTION

BCL-2 protein family members BAK and BAX are the gatekeepers of mitochondrial apoptosis. Homooligomers of BAK and BAX form pores in the mitochondrial outer membrane (MOM) and release apoptotic factors into the cytosol, committing the cell to destruction. Detailed mechanistic studies have revealed how cytosolic and MOM-tethered monomers transform into pore-forming oligomers (*1*). A subset of BH3-only proteins (BOPs) bind BAK and BAX weakly via the canonical hydrophobic cleft shared with prosurvival BCL-2 proteins (*2*, *3*) and, in the case of BAX, also at a proposed alternate “rear pocket” (*4*), These transient interactions trigger a conformational change in BAK or BAX that leads to their homooligomerization. Nuclear magnetic resonance (NMR) and crystallographic models provide views of BAK or BAX as unbound monomer [Protein Data Bank (PDB): 2JCN; PDB: 1F16 (*5*)] and with a BOP bound in the BH3 binding cleft [PDB: 2K7W (*2*); PDB: 2M5B (*4*)]. BAK and BAX are unfolded by detergents, and structures of inert “core-latch” dimers are obtained upon detergent removal. These core-latch dimers adopt a pseudo-monomeric fold, with the α6 to α8 (latch) helices exchanging into a domain-swapped conformation. Crystal structures of these reveal how BOPs bind to partially unfolded BAK and BAX [e.g., PDB: 4U2U (*6*), PDB 4BD2 (*7*)]. Fragments of BAK and BAX have also been crystallized in the active “BH3-in-groove” homodimer conformation, wherein the BH3 domain of each molecule lies in the hydrophobic cleft of the other; this conformation is presumed to nucleate higher-order oligomer formation and assembles a large, exposed hydrophobic surface to enhance MOM interaction [PDB: 4BDU (*7*); PDB: 4U2V (*8*); PDB: 6UXM (*9*)]. Prosurvival BCL-2 proteins block apoptosis by two mechanisms: sequestering BAK- and BAX-activating BOPs and binding the exposed BH3 domain of unfolded BAK or BAX tightly, blocking the formation of BH3-in-groove homodimers.

While many peptide and small-molecule binders exist that target prosurvival BCL-2 proteins (*10*–*16*), no binders are currently available targeting proapoptotic BAK or BAX with both high affinity and specificity (*17*). The design of binders to BAK or BAX is more challenging, partly because they are metastable, with BOP binding resulting in their unfolding, oligomerization, and pore formation to trigger apoptosis, a complication that does not occur for BOP binding to prosurvival BCL-2 proteins. In addition, the transient BH3-mediated interactions of BOPs with the BAK or BAX BH3-binding groove are weaker (high nanomolar or micromolar) than the equivalent interactions with prosurvival BCL-2 family members (low nanomolar) (*18*). Binding prosurvival BCL-2 homologs at their BH3 binding cleft can induce apoptosis; thus, high specificity for BAK or BAX with minimal cross-reactivity with prosurvival homologs is critical for delineating the unique biological contribution of BAK or BAX. Structure-guided mutation of the Bim BH3 peptide yielded a 14.9 nM BAK inhibitor, demonstrating proof of principle that binding with higher affinity than BOPs at BAK’s BH3 binding cleft prevents unfolding and homooligomerization and downstream apoptosis (*6*). However, the inhibitor retains relatively high affinity for prosurvival MCL-1 and BCL-XL (80 and 21 nM, respectively). Mutation and chemical crosslinking of the BCL-2 BH4 sequence yielded a 177 nM BAX inhibitor with unknown cross-reactivity with BAK and prosurvival homologs (*19*). Further development of these molecules is challenging, as peptides are generally poorly cell permeable, and due to incorporation of noncanonical amino acids and chemical crosslinks, they are not genetically encodable and hence cannot be readily incorporated into gene delivery vectors. A small-molecule covalent BAX binder has been generated, but cross-reactivity with BAK and prosurvival homologs is unknown (*20*).

We set out to computationally design genetically encodable protein binders of BAK or BAX with high affinity and specificity. We reasoned that this could be achieved by embedding a BH3 motif in a de novo designed protein, surrounding the motif with secondary structural elements that make additional contacts with BAK and BAX to achieve high affinity and specificity.

## RESULTS

### Computational design yields proteins with low nanomolar binding to BAK and BAX

**Fig. 1.**
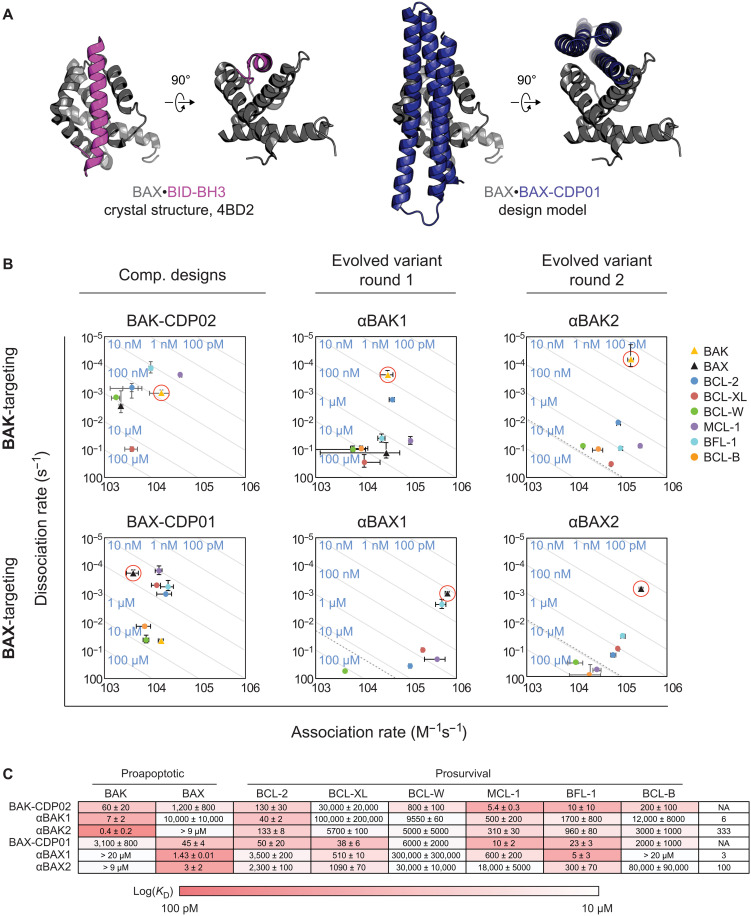
BH3-mimetic designed proteins targeting BAK and BAX. (**A**) BH3 domain of BOP BID interacts with the canonical BH3-binding groove of BAX (PDB ID: 4BD2). The BINDI helix bundle scaffold was docked into the BH3-binding groove of BAX. (**B**) On and off rates were determined by BLI with multiple-concentration binding titrations for each computationally designed protein and optimized variants (means ± SD; *n* = 3). Diagonal lines represent dissociation constants (*K*_d_) as labeled. Dashed lines indicate affinities at which binding signals were too weak to be accurately measured; dissociation constants for interactions not plotted are assumed to be greater than these thresholds. (**C**) *K*_d_ values for CDPs, intermediate, and final optimized variants (means ± SD; *n* = 3).

We previously reported the design and optimization of six three-helix protein inhibitors with high affinity and high specificity for each of seven human and viral prosurvival BCL-2 proteins (*14*, *21*). Although proapoptotic BAK and BAX have opposite function, they are structurally similar and share the canonical hydrophobic BH3-binding cleft. We hypothesized that using similar methods, combining computational protein design and in vitro evolution via yeast surface display (YSD), we could generate potent and specific binders that interact with the BH3-binding cleft of BAK or BAX. While BOPs bind with weak affinity and induce conformational change in BAK and BAX, we hypothesized that designed proteins with very high affinity for BAK and BAX would block the BH3-binding groove, preventing homodimerization and pore formation at the MOM, and ultimately inhibit apoptosis (Fig. 1A).

As a starting point for design, we used as a scaffold a de novo designed protein BINDI that binds with high affinity to BHRF1, a viral prosurvival BCL-2 homolog (*21*). BINDI has high stability (resistance to thermal and chemical denaturation) and therefore is likely to be tolerant of the mutations necessary to enable interaction with BAK and BAX, and we demonstrated previously that the three-helix structure of BINDI can be redesigned for altered BCL-2 family specificity (*14*). Using the Rosetta molecular modeling suite, BINDI was docked into the hydrophobic cleft of BAK and BAX crystallographic, NMR and homology models in which the BH3-binding cleft is in an open conformation. The Rosetta MotifGraft module was used to graft a BH3-like helical motif, including five defined hotspots corresponding to conserved BH3 residues, onto all geometrically compatible positions on the three-helix bundle backbone, generating thousands of variants on tens of unique docked configurations per BAK and BAX model. Rosetta sequence design calculations were carried out on each docked configuration to minimize the free energy of the bound complex. The resulting tens of thousands of designs were filtered on the basis of predicted binding energy, shape complementarity, and number of buried unsatisfied polar atoms at the interface, and 42 (11 targeting BAK and 31 targeting BAX) were selected for experimental characterization.

Genes encoding the designs were expressed in yeast for surface display and screened for binding to recombinant BAK or BAX (*22*). For all designs, computational metrics and yeast binding and expression data are summarized in table S1, and sequences are listed in table S2. To increase recombinant expression and solubility of BAK and BAX, each was expressed in *Escherichia coli* as a fusion to maltose-binding protein (MBP), the N and/or C termini were truncated, and cysteines were mutated to serine. On yeast, 2 of the 11 (18%) BAK-targeting designs bound BAK with moderate affinity, and both also bound BAX with weak or moderate affinity. Two additional BAK-targeting designs exclusively bound BAX with weak affinity. Of the 31 BAX-targeting designs, 14 (45%) bound BAX: two (6%) with high affinity (one also binding BAK with moderate affinity), six (19%) with moderate affinity (one also binding BAK with weak affinity), and six (19%) with weak affinity (one also binding BAK with weak affinity). One additional BAX-targeting design exclusively bound BAK with weak affinity.

Designs with highest affinity and specificity for each target were expressed and purified from *E. coli*, and binding profiles were quantitatively determined with biolayer interferometry (BLI; Fig. 1, B and C). BAK-CDP02 (for BAK-targeting computationally designed protein 02 of 11) bound BAK with 60 ± 20 nM affinity, and BAX-CDP01 (BAX-targeting) bound BAX with 45 ± 4 nM affinity. To compare the designed binders to native interactions with the BH3-binding cleft BAK and BAX, we determined the binding affinity of MBP-fused BH3 peptides from the BOPs BIM and BID. MBP-BIM-BH3 bound BAK and BAX with 4000 ± 2000 nM and 500 ± 100 nM affinity, and BID bound BAK and BAX with >4000 nM and 464 ± 4 nM affinity respectively (table S3). Thus, using computational design alone, we achieved binding affinities two (BAK) and one (BAX) orders of magnitude higher than any known native interaction with the BH3-binding cleft of BAK or BAX.

### Affinity and specificity maturation yield high picomolar to low nanomolar BAK and BAX binders

To delineate the unique biological roles of BAK and BAX, the designed binders must have limited cross-reactivity to structurally similar anti- and proapoptotic homologs. Therefore, we sought to further optimize the partially specific binders for greater specificity and affinity. Site-directed saturation mutagenesis (SSM) was performed on genes for BAK-CDP02 and BAX-CDP01, generating libraries of all possible single amino acid substitutions. Each library was transformed into yeast for surface display and screened via fluorescence-activated cell sorting (FACS) under two conditions: high-affinity binding to labeled target homolog with no competitors and specific binding to labeled target homolog in the presence of unlabeled competitor prosurvival homologs. Naïve libraries and sorted pools were analyzed with next-generation sequencing (NGS) to determine which mutations improved specificity and affinity (figs. S1 and S2). Previous work showed that screening for specificity alone sometimes selects for highly specific variants with weaker on-target affinity than the initial design (*14*) since selection for specificity only requires improvement in the affinity differential of on-target versus off-target homologs. To improve both on-target affinity and specificity simultaneously, we included only those mutants that enriched independently for both affinity and specificity in subsequent combinatorial libraries (fig. S1). Combinatorial libraries were sorted under specificity-enriching conditions six times, with decreasing concentration of target homolog and/or increasing concentration of competitors (SSM and combinatorial library design and selection conditions in tables S4 and S5). Variants enriched in the final sort of each library were expressed in *E. coli* and purified, and their binding profiles were qualitatively compared with single-concentration BLI experiments.

The best variants from each library, αBAK1 and αBAX1, exhibited on-target affinity an order of magnitude greater than their precursors (Fig. 1C). The optimized variants had much weaker affinity for most off-target homologs, but each retained high affinity binding to one prosurvival homolog: αBAK1 bound BCL-2 with 40 ± 2 nM affinity and αBAX1 bound BFL-1 with 5 ± 3 nM affinity. αBAK1 differs from its CDP precursor by 10 mutations (8%) and αBAX1 by 8 mutations (7%; see table S5 for summary of mutations). To further enhance specificity and affinity, a second round of in vitro evolution was performed, again including in the second-generation combinatorial libraries only those mutants of αBAK1 or αBAX1 that improve both affinity and specificity. Five rounds of FACS under specificity-enriching conditions yielded binders with high affinity and specificity. αBAK2 binds BAK with 400 ± 200 pM affinity and 333-fold specificity (Fig. 1C). αBAX2 binds BAX with 3 ± 2 nM affinity and 100-fold specificity. αBAK2 differs by 12 mutations (10%) from precursor αBAK1 and 22 mutations (19%) from BAK-CDP02. αBAX2 differs by 10 mutations (9%) from precursor αBAX1 and 18 mutations (15%) from BAX-CDP01. Nearly all mutations made from the precursor variants to yield the final variants were depleted in the CDP-based SSM, indicating that most mutations made in the second round of evolution are context dependent (fig. S1, C and F). Each protein exhibits cooperative unfolding when exposed to increasing concentrations of denaturant, suggesting a well-packed core, although denaturation midpoints shift lower with each round of evolution indicating the new mutations destabilize the fold (fig. S3).

Several structural features likely contribute to the remarkable greater than 100-fold specificity of the optimized designs for BAK and BAX over other BCL-2 family members. The loop between helices 4 and 5 (α4 and α5) of the BCL-2 homologs, positioned at the periphery of the BH3-binding interface, is one residue longer for BAX, BFL-1, and MCL-1 than BAK, BCL-2, BCL-XL, and BCL-W (fig, S4, A and C). On the other side of the interface, at the segment spanning α2 and α3 (beginning with the C-terminal segment of the BH3 motif), BAK is most similar to BCL-2 at key interface residues, while BAX is most similar to MCL-1 and BFL-1 (fig. S4B). The α4 and α5 helices are structurally similar among the eight homologous proapoptotic and prosurvival proteins. While the α5 sequence is largely conserved likely due to its burial in the hydrophobic core, the α4 sequence varies considerably, giving each homolog a unique electrostatic profile at key interface positions (fig. S4C). Evolved αBAX2 residue K95 and designed residue E94 likely improve specificity by taking advantage of sequence differences in this region; opposing BAX residue D98 (sequence position 9 in fig. S4C, modeled in fig. S4D) complements evolved αBAX2 residue K95 and BAX R94 (fig. S4C, position 5) complements designed αBAX2 residue E94, while at least one of the two analogous positions of every other homolog has a different residue type.

### Crystal structures of the BAK:αBAK2 complex agree with the design model

**Fig. 2.**
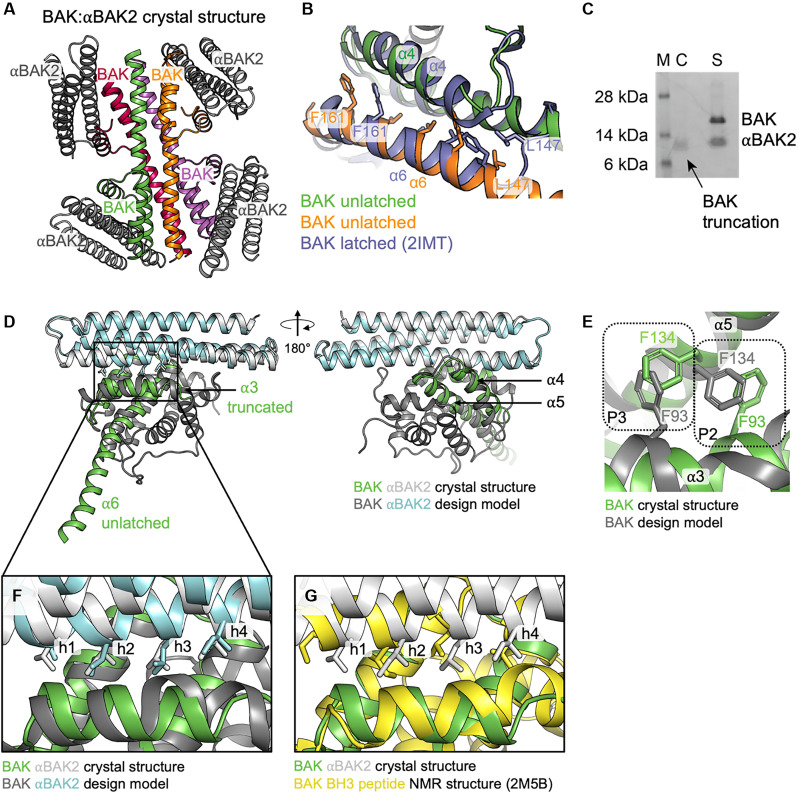
Crystal structure of the BAK:αBAK2 complex. (**A**) Crystal structure asymmetric unit containing four copies of BAK:αBAK2 heterodimer complexes with BAK in color (green, orange, red, and magenta) and αBAK2 in gray. (**B**) Comparison of the BAK α6 helix interactions with α4 and α5 helices for a single BAK monomer (slate, PDB ID: 2IMT) and the crystal contact between neighboring BAK molecules (green and orange) in the BAK:αBAK2 crystal structure. Positions of equivalent residues from L147 to F161 are shown indicating a register shift of one helical turn. (**C**) SDS-PAGE showing BAK protein is truncated in the crystals (C) but not in solution (S), and protein standard (M) sizes are indicated. (**D**) A single BAK:αBAK2 heterodimer from the crystal structure (green and gray) aligned to the BAK:αBAK2 design model (dark gray and cyan) shows that the designed binding mode closely matches the crystal structure. In the crystal structure, BAK α3 is truncated and α6 and α7 form an extended helix in an “unlatched” conformation. (**E**) BAK residues F93 and F134 that line the BAK hydrophobic pocket change rotamers to accommodate the register shift of α3 by one helical turn in the crystal structure compared to design. (**F**) Interactions between αBAK2 and BAK show that the BH3-mimetic hydrophobic residues of αBAK2 (h1-4) occupy the same BAK binding pockets in design and crystal structure. (**G**) BAK-binding mode of αBAK2 is similar to that of a BH3 peptide.

We solved a structure of the BAK:αBAK2 complex in the P1 space group, diffracting to 2.9 Å, with four BAK:αBAK2 heterodimers in the asymmetric unit (Fig. 2A and table S6). The four heterodimeric complexes in the asymmetric unit are similar (between 0.15- and 0.29-Å Cα root mean square deviation (RMSD), 193 to 195 atoms aligned from a total of 197). BAK is in an unlatched conformation with α6 to α8 helices dissociated from the BAK monomer core. The unlatched α6 and α7 helices form a tetrameric BAK crystal contact in the structures, with their hydrophobic faces packing against α4 and α5 helices from a neighboring BAK monomer (Fig. 2B). In previous structures of the BAK monomer and of a BAK “core-latch” domain swap dimer, the α6 helix also packs against the α4 and α5 helices (*8*, *23*). However, the α6 helix shifts register by one helical turn in the BAK:αBAK2 structure relative to these BAK monomer and domain swap dimer structures. This crystal contact is seen in the detergent-activated BAK structure (PDB: 7K02), where the α6 and α7 helices are also unlatched upon activation with detergent (*24*). No electron density is seen for the BAK α1 and α2 helices. The SDS–polyacrylamide gel electrophoresis (SDS-PAGE) of the crystals shows that the BAK construct is truncated, but only in the crystals and not in solution (Fig. 2C). This indicates that αBAK2 can bind to an unfolded BAK in the BH3 binding groove.

The design model of BAK:αBAK2 is very close to the crystal structure, with an all-atom RMSD of 3.2 Å and Cα RMSD of 2.6 Å after alignment of BAK α3 to α5 helices (residues 90 to 141) and αBAK2 (Fig. 2D). BAK structural elements further from the binding interface differ due to the conformational change in BAK α6 and α7 helices, and the truncation eliminating α1 and α2 helices described above. The position of BAK helix α3 relative to helices α4 and α5 is shifted by approximately one helical turn in the crystal structure compared to the design model, which was based on NMR structure 2M5B. BAK residues F93 (α3) and F134 (α5) change rotamers to accommodate this shift, with F93 in the crystal structure inserting into the BAK P2 pocket occupied by F134 in the design model and vice versa with F134 in the crystal structure inserting into the BAK P3 pocket occupied by F93 in the design model (Fig. 2E). Despite the α3 shift, the αBAK2 h1-h4 BH3-mimetic residues occupy similar positions at the BAK interface relative to BAK α4 and α5 helices (Fig. 2F). The design model accurately captures the shift in binding mode of αBAK2 compared to a BH3 peptide bound to BAK in NMR structure 2M5B, which the structure design was based on (Fig. 2G). We also solved a crystal structure of unbound αBAK2 at 2.8-Å resolution (table S6 and fig. S5). The two molecules present in the asymmetric unit have similar structures (0.62-Å Cα RMSD) and both globally agree with the design model (1.3- and 1.1-Å Cα RMSD) and the αBAK2 monomer in the cocrystal structure (1.2- and 1.9-Å Cα RMSD; fig. S5A), with some deviation at the N-terminal end of the second helix (fig. S5B). In conclusion, αBAK2 binds as designed in the BAK BH3-binding groove and can bind to BAK in a partially unfolded, unlatched conformation.

### αBAK2 and αBAX2 activate or inhibit BAK and BAX, depending on relative concentration

**Fig. 3.**
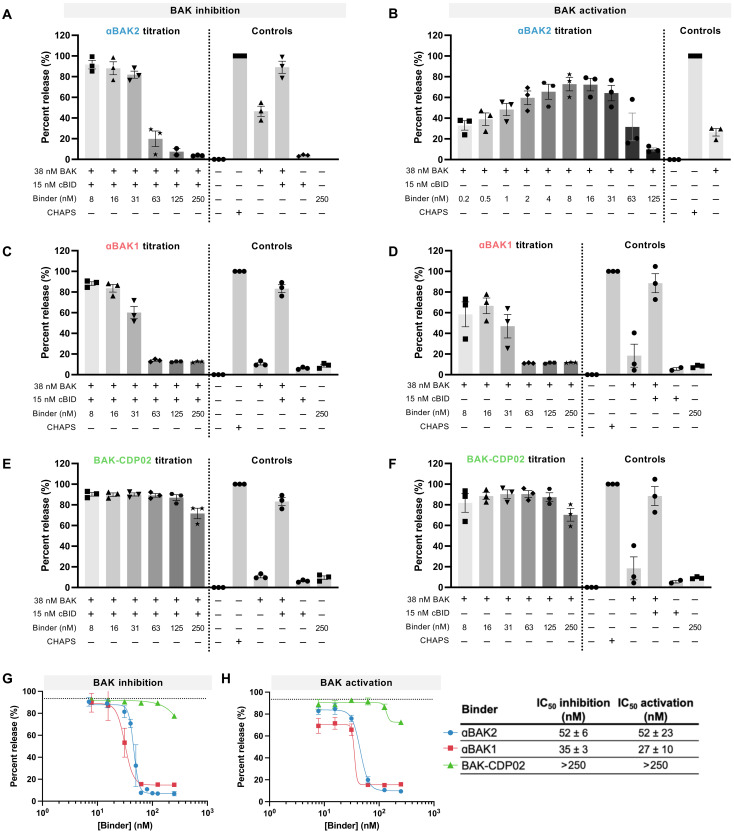
BAK binders activate or inhibit BAK, depending on their binding affinity and relative concentration. (**A**, **C**, and **E**) Liposomes were treated with BAK, an activating concentration of BOP cBID, and a titration of each binder to determine their propensity to inhibit BAK-mediated permeabilization or (**B**, **D**, and **F**) treated with BAK and a titration of binder to determine their propensity to activate BAK-mediated permeabilization. The fluorescence intensity of the assay solution, as a proxy for liposome permeabilization, was monitored over time, and the end-point release percent was plotted relative to the positive control treatment (CHAPS). (**G** and **H**) End-point release percent was plotted versus binder concentration. Dashed lines indicate the percent release of control treatment without binder (activation: BAK only; inhibition: BAK + cBID). [(A) to (F)] Bars represent mean values with an SE from three independent experiments shown with symbols, with each independent experiment performed in technical triplicate. [(G) and (H)] Representative IC_50_ curves showing mean with SD as error from technical triplicates. IC_50_ values are presented as the means ± SD from the three independent experiments shown in (A) and (B).

**Fig. 4.**
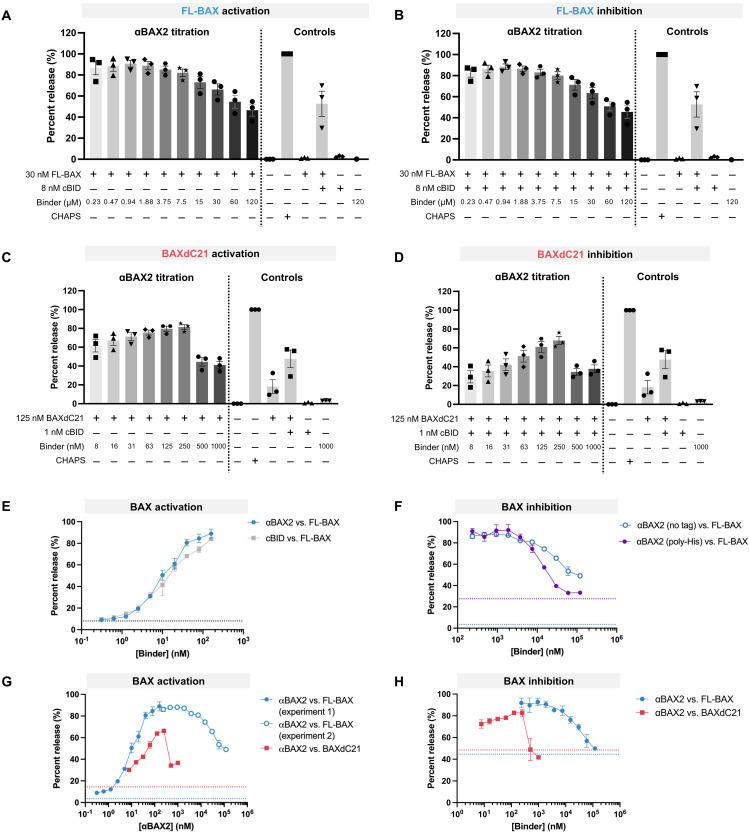
BAX binders activate or inhibit BAX, depending on their binding affinity and relative concentration. (**A** and **C**) Liposomes were treated with full length BAX (FL-BAX) or BAX lacking the C-terminal transmembrane domain (BAXdC21) and a titration of αBAX2 to determine its propensity to activate BAX-mediated permeabilization or (**B** and **D**) an activating concentration of BOP cBID and a titration of αBAX2 to determine its propensity to inhibit BAX-mediated permeabilization. Fluorescence intensity of the assay solution, as a proxy for liposome permeabilization, was monitored over time, and end-point release percent was plotted relative to the positive control treatment (CHAPS). (**E**) End-point release percent was plotted versus binder concentration for αBAX2 (blue) and cBID (gray). (**F**) End-point release percent was plotted versus binder concentration for αBAX2 with no his tag (blue) and αBAX2-his (purple) with liposomes containing an NTA-DOGS lipid. (**G** and **H**) End-point release percent was plotted versus binder concentration. Dashed lines indicated the percent release of control treatment without binder (activation: BAX only; inhibition: BAX + cBID; blue lines FL-BAX, red lines BAXdC21). [(A) to (D)] Bars represent mean values with SE from three independent experiments shown with symbols, with each independent experiment performed in technical triplicate. [(E) and (F)] Representative dose-response curves showing the means ± SD from technical triplicates, from the one independent experiment shown in fig. S10. [(G) and (H)] Representative dose-response curves showing the means ± SD from technical triplicates.

BAK and BAX form pores in the mitochondrial membrane that allows cytochrome *c* to enter the cytosol, a critical step in apoptosis. We used established liposome permeabilization assays to interrogate how αBAK2 and αBAX2 affect this function (Figs. 3 and 4) (*7*, *8*). In these assays, when BAK or BAX are activated (e.g., with the BOP BID or heat treatment), they dimerize and form pores in the liposome membranes, causing release of self-quenching dye from the liposomes into solution. Increased fluorescence detected in solution is therefore a proxy for BAK or BAX activation. When C-terminally truncated BAK (BAKdC25 with a C-terminal 6-histidine tag) is activated with cleaved BID (cBID) or heat treatment (43°C) and treated with a titration of αBAK2, a dose response is observed: At the lowest tested concentrations, αBAK2 shows no impact on cBID- or heat-mediated BAK activation, but as αBAK2 concentration increases, the inhibition of BAK dimerization correspondingly increases until permeabilization is limited to the levels seen for unactivated BAK control (Fig. 3A and figs. S6A and S7A). Unexpectedly, in the absence of the activator, lower concentrations of αBAK2 induce BAK activation comparable with cBID (Fig. 3B and fig. S7, B to D). However, as increasing αBAK2 concentrations saturate the BAK pool, unlike cBID, αBAK2 does not achieve complete release and inhibits αBAK2-mediated activation. Furthermore, when cBID is included in the assay at a fixed concentration (15 nM), titrating αBAK2 showed a similar inhibition profile to when cBID is absent (Fig. 3, A and B, and fig. S7A). The αBAK2 dose-response curves are very similar in the presence and absence of cBID, with median inhibitory concentration (IC_50_) values of αBAK2 (51.9 and 51.6 nM, respectively) very close to the molar concentration of BAK used in the assay (38 nM; Fig. 3, A, G, and H). When αBAK2 was added several minutes after BAK activation was induced by cBID, αBAK2 blocked further permeabilization (fig. S7E), and incubating αBAK2 with the permeabilized liposomes resulted in no change in BAK oligomer bands (fig. S8). Thus, αBAK2 shows a concentration threshold where it switches from inducing pore formation to inhibiting it and that further pore formation can be blocked after it has initiated, indicating that pores formed on other liposomes cannot rupture other intact liposomes.

Treating full-length BAX (FL-BAX) with both cBID and αBAX2 resulted in increased liposome permeabilization compared to FL-BAX treatment with αBAX2 alone (Fig. 4, A and B, and fig. S9, A and B). Treating FL-BAX with αBAX2 showed liposome permeabilization at low nanomolar concentrations comparable with cBID alone, indicating that it is a potent activator of BAX (Fig. 4, A and E, and fig. S10, A and B). As αBAX2 concentration increased, αBAX2-mediated BAX activation was partially inhibited. The αBAX2 concentration required to inhibit BAX can be reduced by targeting αBAX2 to liposomes with a C-terminal his tag (αBAX2-his) that associates with a nitrilotriacetic acid (NTA)–lipid in the membrane, despite FL-BAX showing more auto-activity with this lipid composition (Fig. 4F and fig. S10C). This infers that the localization of αBAX2 is important to prevent BAX oligomerization. While a simple molar excess is required for inhibition of BAK by αBAK2 (Fig. 3), the inhibition of FL-BAX requires a ~1000-fold molar excess of αBAX2 (Fig. 4, B and F). This observation is not likely explained by differences in binding affinity alone, as αBAX2 [dissociation constant (*K*_d_) 3 nM) has only eightfold weaker affinity for BAX than αBAK2:BAK (*K*_d_ 400 pM). BAX, but not BAK, is predominately cytosolic with its C-terminal helix (α9) bound in the BH3-binding groove. The BAX α9 competes with BOPs and is predicted to compete with αBAX2 (based on the design model) for binding to BAX and thus may raise the concentration of αBAX2 required for BAX inhibition. When the same experiments were performed with a truncated version of BAX lacking α9 (BAXdC21 with a C-terminal 6-histidine tag), αBAX2 inhibits BAXdC21 activation when only a two- to fourfold molar excess of αBAX2 is reached (Fig. 4, C and G, and fig. S9C). In the absence of cBID, similar trends occur (Fig. 4, D and H, and fig. S9D). These data indicate that the presence of the BAX α9 indeed influences the consequent activity of αBAX2 binding.

### BAK-binding affinity alters functional outcomes

We sought to determine the impact of binding affinity on the activating versus inhibiting behavior of the BAK binders. During the design and evolution of αBAK2, we generated two lower affinity constructs for BAK, αBAK1, and CDP02, which bound to BAK with *K*_d_ values of 7 and 60 nM respectively. In liposome assays, αBAK1 shows similar behavior as αBAK2 (Fig. 3, C and D, and fig. S6, C and D). In contrast, CDP02 shows only a small amount of BAK inhibition at the highest concentration tested (Fig. 3, E and F, and fig. S6, E and F). These data indicate that the high affinity achieved for αBAK2 and αBAK1 is required to inhibit BAK pore formation, and reducing this affinity increases the molar excess of binder required for inhibition. Conversely, high affinity binding is not a necessary requirement for BAK activation.

### αBAK2 and αBAX2 induce or inhibit cytochrome *c* release from mitochondria, depending on concentration

**Fig. 5.**
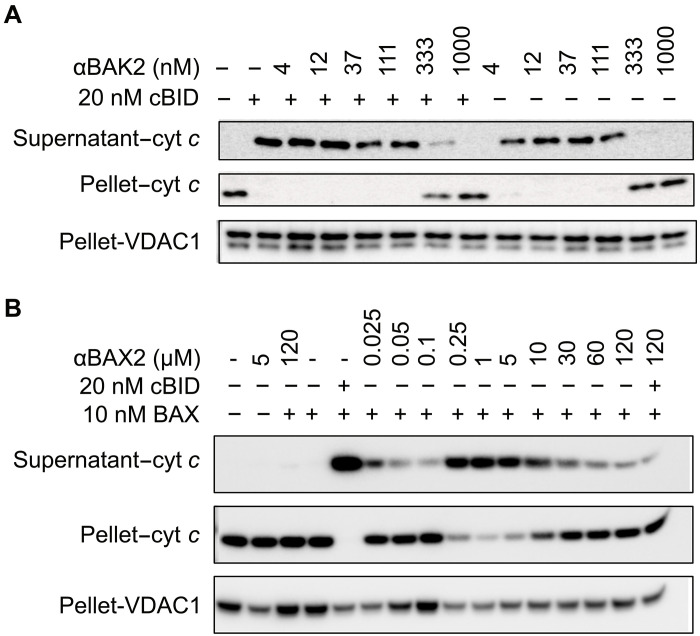
αBAK2 and αBAX2 induce or inhibit cytochrome *c* release from mitochondria, depending on their concentration. Consistent with liposome permeabilization data, in a concentration-dependent manner (**A**) αBAK2 induces or inhibits BAK activation and subsequent release of cytochrome *c* from the mitochondrial intermembrane space of MEFs expressing human BAK, and (**B**) αBAX2 induces or inhibits BAX activation and cytochrome *c* release from mouse liver mitochondria treated with recombinant BAX. BID serves as a positive control to activate BAK and BAX. Blots were reblotted for VDAC1 as a loading control. Blots are representative of three independent experiments.

We next sought to test whether the binders could affect apoptotic signaling in the cellular milieu. αBAK2 binds with high affinity to human BAK but does not cross-react with the mouse BAK ortholog. mBAK and mBAX double knockout mouse embryonic fibroblasts (MEFs) were engineered with constitutive expression of full-length human BAK (FL-BAK) to test the apoptotic signaling of αBAK2 (Fig. 5A and fig. S11). When permeabilized cells are treated with cBID, FL-BAK is activated and forms pores in the mitochondrial membrane, causing the release of cytochrome *c* from the intermembrane space as observed in Western blots where cytochrome *c* transitions from the mitochondria-containing pellet to cytosol-containing supernatant fractions. In agreement with liposome data, αBAK2 alone induced cytochrome *c* release at lower concentrations but not at higher concentrations (≥333 nM). Cotreatment with cBID and αBAK2 inhibited cytochrome *c* release upon increasing αBAK2 concentration, with complete inhibition observed at 1 μM. This shows that αBAK2 can inhibit pore formation and cytochrome *c* release from mitochondria upon an apoptotic stimulus. Notably, higher αBAK2 concentrations were required for inhibition of cytochrome *c* release compared to inhibition of liposome permeabilization. This could be due to different molecular contexts, such as BAK concentration, which is defined in liposome assays but not in cytochrome *c* release assays where it is overexpressed, or the presence of prosurvival proteins, although previous studies have found that BAK does not form these complexes in these assays unless the prosurvivals are overexpressed (*25*, *26*). In addition, the recombinant BAK used in liposome assays lacks the α9 helix, whereas expressed BAK in the cytochrome *c* release assays is full length. Notably, BAK α9 helix stabilizes its interaction with VDAC2 and inhibits BAK activation. This interaction is of particular interest as VDAC2-BAK interaction helps tumor cells resist immune checkpoint inhibitors. (*27*)

The behavior of αBAX2 in a cellular environment was tested using mitochondria from the livers of an mBAK knockout mouse (Fig. 5B and fig. S12). Mitochondria were isolated, removing endogenous cytosolic mBAX and reconstituted in media containing recombinant human FL-BAX. When treated with cBID, all cytochrome *c* moved to the supernatant, indicating FL-BAX activation and pore formation. Consistent with liposome assays, a high concentration of αBAX2 (120 μM) inhibits cBID-mediated activation of BAX. In the absence of cBID, αBAX2 showed concentration-dependent activation or inhibition of BAX-mediated cytochrome *c* release, consistent with liposome data. Further, in this assay, αBAX2 (120 μM) shows a similar loss of cytochrome *c* release in the presence and absence of cBID.

## DISCUSSION

We describe potent and selective designed protein binders of the human proapoptotic BCL-2 proteins BAK and BAX, with high picomolar to low nanomolar affinity, orders of magnitude stronger than any existing binders. Specificity is critical for agents to be used as molecular probes or therapeutic modulators of apoptosis; to our knowledge, our designed proteins are the only BAK or BAX groove binders with high specificity, having greater than 100-fold weaker affinity for prosurvival BCL-2 homologs. Crystal structures agree with the computational model and confirm the BH3-mimetic binding mode of αBAK2 with BAK.

**Fig. 6.**
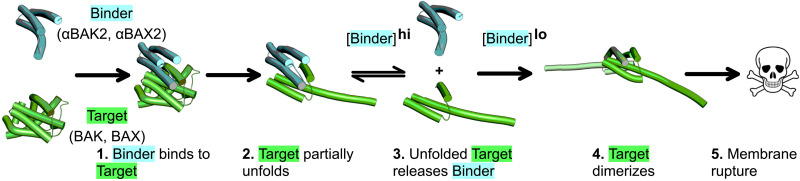
Schematic describing proposed mechanism of BAK/BAX activation and inhibition by designed binders. (1) Designed binders (αBAK2 or αBAX2) bind to their respective proapoptotic effector target (BAK or BAX). (2) Binders induce a conformational change that partially unfolds the target. (3) Outcomes are specific to the concentration of binder relative affinity: At relatively low binder concentrations, the target is activated, dimerizes, and forms pores (4 and 5), while at relatively high binder concentrations, the binders saturate the target pool and inhibit dimerization, preventing pore formation (2).

The designed binders unexpectedly act as both activators (at relatively low concentrations) and inhibitors (at relatively high concentrations). We propose that upon binding, BAK or BAX unfold into “activated” monomers due to their inherent metastability. When the concentration of binder is sufficient to saturate the pool of activated BAK/BAX monomers, dimerization is inhibited, and the membrane remains intact. However, if binder concentration is not sufficient to saturate the activated BAK/BAX pool, then dimers form, and the membrane is ruptured (Fig. 6). The threshold concentration of binder required to saturate the pool is dependent on the affinity for the BAK/BAX groove. 

The BAK and BAX binders have different activation and inhibition thresholds for their targets. While αBAX2 has a slightly weaker affinity for BAX than αBAK2 has for BAK, this does not fully explain the differences in inhibition and activation potency. This difference may be explained by the distinct role for the α9 helix between BAK and BAX. BAX is predominately in the cytosol with the α9 helix buried in the groove, while BAK is mostly at the MOM with the α9 helix inserted into the membrane. A binder needs to compete with the BAX α9 helix to inhibit, but this exposes the hydrophobic α9 helix facilitating membrane insertion and may contribute to BAX activation. In contrast, no competition with the α9 helix is required for binding to the BAK groove as the α9 helix is already inserted into the MOM.

The threshold concentrations for activation versus inhibition of full-length BAK and BAX by αBAK2 and αBAX2 should be considered when using the designed binders as probes or therapeutics in situ. αBAK2 may be usable as a BAK inhibitor in situ, but αBAX2 will not be, as the concentrations required to saturate the BAX pool are likely not feasible. Despite this, αBAX2 can be used as a selective BAX activator in situ as it does not bind to prosurvival proteins or BAK. The same is true for αBAK2; because of its potency as an activator, it is likely to induce apoptosis if expressed in cells without due care to maintain high expression levels with no ramp up in concentration. This work demonstrates that binding the BH3 groove is a reasonable approach to BAK inhibition and could inform small-molecule design. In contrast, BAX inhibition by binding the BH3 groove is likely intractable for small molecules as a low picomolar *K*_d_ would likely be required, and alternate BAX sites may therefore be better targets for inhibition.

The designed BAK and BAX binders provide a new route to selectively modulate apoptosis in medicine and cell engineering applications. Current methods to inhibit apoptosis in vitro, such as caspase inhibition, act downstream of the apoptotic “point of no return,” i.e., BAK- and BAX-mediated pore formation in the MOM. Blocking apoptosis further upstream at BAK and BAX should maintain MOM integrity and thus protect more broadly against both caspase-dependent and -independent cell death associated with MOM permeability (*28*, *29*). The BAX selective activators allow specific induction of BAX-mediated apoptosis, allowing disentanglement of the BAK and BAX pathways without the need for knockouts. In addition, the BAX activators could be engineered as a kill switch for cellular therapies, allowing specific induction of BAX-mediated apoptosis upon abhorrent function, e.g., cytokine storm or tumor lysis syndrome from cellular therapies expressing chimeric antigen receptors. Designed apoptosis inhibitors could address current bottlenecks limiting the production of engineered cells for adoptive therapy: poor ex vivo viability, recovery after genetic modification, and efficient selection and expansion of modified cells. Our designed binders have the specificity and potency required for efficient modulation apoptosis via a direct interaction with BAK and BAX and provide the proof of concept required to address these challenges.

## MATERIALS AND METHODS

### Computational methods

#### General methods

Rosetta software can be downloaded from www.rosettacommons.org and is available free to academic users. Online documentation can be found at https://docs.rosettacommons.org/manuals/latest/, and instructions for RosettaScripts syntax is available at www.rosettacommons.org/docs/latest/scripting_documentation/RosettaScripts/RosettaScripts.

A comprehensive list of command line options for Rosetta can be found at www.rosettacommons.org/docs/latest/full-options-list. All computational protocols were executed from within the RosettaScripts framework, which enables the user to piece together select portions of Rosetta code to generate project-specific protocols (*30*, *31*). A basic example of a command line executed to launch ROSETTA using a RosettaScripts protocol is as follows

/path/rosetta_scripts.default.linuxgccrelease

–database /path/main/database

–parser:protocol rosetta_scripts_protocol.xml

-in:file:s input.pdb

RosettaScripts XML protocols can be found below. The Rosetta score function Talaris13 was used for all experiments.

#### Input model selection and generation

Our goal was to de novo design a helix bundle protein to bind BAK or BAX at the canonical BH3-binding cleft, such that the designed protein mimics the interaction of a helical BH3-motif with BAK and BAX but additionally expands the interaction surface to support affinity- and specificity-enhancing contacts. Crystal and NMR structures show that the BH3-binding cleft takes on a slightly more open conformation when bound to a BH3 helix. We therefore chose to only use helix-bound structures and models of BAK and BAX as inputs for design. The following crystallographic and NMR models of BAK and BAX were used as input for computational experiments described below: 1F16, monomeric BAX with its own C terminus occupying the canonical binding cleft (*5*); 4BD2, BAX domain-swapped dimer in complex with BID-BH3 (*7*); 4BD6, BAX domain-swapped dimer in complex with BAX-BH3 (*7*); and 2M5B, BAK in complex with BID-BH3 (*2*).

As a scaffold for inhibitor design, we used the helix bundle BINDI, a designed inhibitor of BHRF1, a viral prosurvival BCL-2 homolog (*21*). BINDI has high stability, i.e., resistance to thermal and chemical denaturation, and therefore is likely to be more tolerant of the mutations necessary to enable interaction with BAK and BAX. After alignment of the BAK or BAX structures above with the structure of BINDI-bound BHRF1, the bound peptides (either BH3 domains or BAX’s own N terminus) were replaced with backbone and beta carbon atoms of BINDI residues spanning the central binding helix (I50-N62; 4OYD); the backbone conformation and position of bound peptides in this region is very consistent among crystal structures of BH3-bound homologs and BINDI-like inhibitor-bound homologs as in PDB IDs 4OYD, 5JSB, and 5JSN (*14*).

Additional models of helix-bound BAK and BAX were generated using the TM-align software (*32*). To predict the conformation of BAK and BAX bound to the BINDI helix bundle scaffold, BAK and BAX sequences were structurally aligned with the crystal structure of BHRF1, a viral prosurvival homolog, bound to the designed helix bundle inhibitor BINDI (4OYD).

For all models generated above, the identities of five residues that are conserved among BH3 domains and believed to be critical for binding the canonical hydrophobic cleft were kept constant: BINDI residues I50, L54, I57, G58, and D59, corresponding to positions h1, h2, h3, h3 + 1 and h3 + 2, respectively. All other residues of the modeled BINDI-BH3 motif were mutated to alanine. Each BAK·BINDI-BH3 and BAX·BINDI-BH3 complex then underwent constrained backbone and side-chain minimization before input as “context” (BAK or BAX) and “motif” (bound peptide) in MotifGraft.

#### Computational docking of a stable scaffold in the BH3-binding cleft of BAK and BAX

The Rosetta MotifGraft module was used to generate docked conformations of BINDI, a de novo designed inhibitor of BHRF1, in the BH3-binding groove of BAK and BAX and has been described previously (*14*). Briefly, a motif (the mutated helical BINDI-BH3 peptide) is embedded into a larger protein scaffold (BINDI, a three-helix bundle) at any position on the scaffold protein that is geometrically compatible. A representative RosettaScripts protocol executing the MotifGraft module and downstream filtering steps is briefly annotated below. Please see detailed documentation at www.rosettacommons.org for more information regarding each item (TaskOperations, Movers, Filters).

<ROSETTASCRIPTS>

**TaskOperations define subsets of residues such that modifications specified below (Movers) can be restricted to the desired subset(s).**

<TASKOPERATIONS>

<InitializeFromCommandline name=“init”/>

<LimitAromaChi2 name=“arochi2”/>

<IncludeCurrent name=“inclcur”/>

<ExtraRotamersGeneric name=“exrot” ex1=“1” ex2=“1” 

extrachi_cutoff=“1”/>

<OperateOnCertainResidues name=“hotspot_norepack”>

<PreventRepackingRLT/>

<ResiduePDBInfoHasLabel property=“HOTSPOT”/>

</OperateOnCertainResidues>

<OperateOnCertainResidues name=“scaffold_norepack”>

<PreventRepackingRLT/>

<ResiduePDBInfoHasLabel property=“SCAFFOLD”/>

</OperateOnCertainResidues>

<RestrictToInterface name=“interface_12A” jump=“1” distance=“12.0”/>

<RestrictToRepacking name=repack_only />

</TASKOPERATIONS>

**Movers define modifications to the input structure.**

<MOVERS>

<MotifGraft name=“motifgraft” context_structure=“context.

pdb” motif_structure=“motif.pdb” RMSD_tolerance=“3.0” NC_points_RMSD_tolerance=“2.0” clash_score_cutoff=“0” clash_test_residue=“GLY” hotspots=“1:5:8:9:10” full_motif_bb_alignment=“1” revert_graft_to_native_sequence=“1”/>

<TaskAwareMinMover name=“minimization” bb=“0” chi=“1” 

task_operations=“interface_12A,init,inclcur,arochi2,exrot”/>

<PackRotamersMover name=“repacking” 

task_operations=“interface_12A,init,inclcur,arochi2,exrot,repack_only,hotspot_norepack,scaffold_norepack”/>

<ParsedProtocol name=“minimization_repacking_minimization”>

<Add mover=“minimization”/>

<Add mover=“repacking”/>

<Add mover=“minimization”/>

</ParsedProtocol>

</MOVERS>

**Filters define computed values that the modified input structure must achieve in order to be considered successful.**

<FILTERS>

<Geometry name=“omega_filter” omega=“150” cart_bonded=“30” 

confidence=“1”/>

</FILTERS>

**Protocols implement the movers and filters defined above.**

<PROTOCOLS>

**MotifGraft aligns the scaffold onto the bound motif and generates a model of the scaffold in complex with the context (BAK or BAX).**

<Add mover=“motifgraft”/>

**The resulting scaffold:context complex is relaxed.**

<Add mover=“minimization_repacking_minimization”/>

**The relaxed scaffold:context complex is filtered for acceptable incorporation of the motif into the scaffold, specifically checking that peptide bond omega geometries are normal.**

<Add filter=“omega_filter”/>

</PROTOCOLS>

</ROSETTASCRIPTS>

#### Sequence design and scoring

Each of the thousands of docked conformations of BINDI in the BH3-binding cleft of BAK and BAX was then input into a RosettaScripts protocol for sequence design. Rosetta Monte Carlo sequence design calculations were carried out at BINDI surface residues at the BAK or BAX surface. This stochastic sampling method optimizes side-chain identities at the selected positions that minimize the free energy of the bound complex. The more favorable the free energy of the bound complex relative to the free energy in the unbound state, the higher the theoretical affinity of the binding interaction. Consecutive sequence design calculations were made first at BINDI residues within 8 Å of the BAK or BAX surface and then at residues within 12 Å, followed by rigid-body minimization. BINDI residues in the hydrophobic core and surface residues further than 12 Å from the BAK or BAX surface were not allowed to mutate. Metrics associated with design success were calculated for each of the thousands of output models of designed binders in complex with BAK or BAX.

<ROSETTASCRIPTS>

**TaskOperations define subsets of residues such that modifications specified below (Movers) can be restricted to the desired subset(s).**

<TASKOPERATIONS>

<InitializeFromCommandline name=“init”/>

<LimitAromaChi2 name=“arochi2”/>

<IncludeCurrent name=“inclcur”/>

<ExtraRotamersGeneric name=“exrot” ex1=“1” ex2=“1” extrachi_cutoff=“1”/>

<OperateOnCertainResidues name=“hotspot_norepack”>

<PreventRepackingRLT/>

<ResiduePDBInfoHasLabel property=“HOTSPOT”/>

</OperateOnCertainResidues>

<OperateOnCertainResidues name=“scaffold_norepack”>

<PreventRepackingRLT/>

<ResiduePDBInfoHasLabel property=“SCAFFOLD”/>

</OperateOnCertainResidues>

<SelectBySASA name=“surface” mode=“sc” state=“monomer” probe_radius=“2.2” core_asa=“0” surface_asa=“30” core=“0” boundary=“0” surface=“1”/>

</TASKOPERATIONS>

**Movers define modifications to the input structure.**

<MOVERS>

<RepackMinimize name=“design1_8A” repack_partner1=“1” repack_partner2=“1” design_partner1=“0” design_partner2=“1” interface_cutoff_distance=“8.0” minimize_bb=“0” minimize_rb=“0” minimize_sc=“1” task_operations=“init,inclcur,arochi2,exrot,surface,dont_allow_PCWG,rtr_hotspots”/>

<RepackMinimize name=“design2_12A” repack_partner1=“1” repack_partner2=“1” design_partner1=“0” design_partner2=“1” interface_cutoff_distance=“12.0” minimize_bb=“0” minimize_rb=“0”minimize_sc=“1” task_operations=“init,inclcur,arochi2,exrot,surface,dont_allow_PCWG,rtr_hotspots”/>

<TaskAwareMinMover name=“minimization” bb=“0” chi=“1” task_operations=“interface_12A,init,inclcur,arochi2,exrot”/>

<PackRotamersMover name=“repacking” task_operations=“interface_12A,init,inclcur,arochi2,exrot,repack_only,hotspot_norepack,scaffold_norepack”/>

<ParsedProtocol name=“minimization_repacking_minimization”>

<Add mover=“minimization”/>

<Add mover=“repacking”/>

<Add mover=“minimization”/>

</ParsedProtocol>

</MOVERS>

**Filters define computed values that the modified input structure must achieve in order to be considered successful.**

<FILTERS>

<Ddg name=“binding_energy” scorefxn=“talaris2013” threshold=“%%ddg_threshold%%” confidence=“1”/>

<ShapeComplementarity name=“shape_complementarity” min_sc=“%%Sc_threshold%%” confidence=“1”/>

<BuriedUnsatHbonds name=“unsatisfied_hbond_atoms” cutoff=“%%unsat_hreshold%%” confidence=“1”/>

</FILTERS>

**Protocols implement the movers and filters defined above.**

<PROTOCOLS>

**Sequence design modifies the BINDI scaffold sequence to promote binding to BAK or BAX.**

<Add mover=“design1_8A”/>

<Add mover=“design1_12A”/>

**The design complex is relaxed.**

<Add mover=“minimization_repacking_minimization”/>

**Filters are used to score each design.**

<Add filter=“binding_energy”/>

<Add filter=“shape_complementarity”/>

<Add filter=“unsatisfied_hbond_atoms”/>

</PROTOCOLS>

</ROSETTASCRIPTS>

### Recombinant protein expression and purification

Computationally designed proteins were codon-optimized for *E. coli*, commercially synthesized by Gen9, and cloned into pET29b for expression in *E. coli*. For use in in vitro binding and mechanism assays and directed evolution via YSD, genes encoding proapoptosis homologs (BAK 16-186 and BAX 1-166 C62S C126S) were codon-optimized for expression in *E. coli* and cloned into the pMAL-c5x plasmid for N-terminal fusion to MBP and modified at the C terminus with either a 6-histidine tag for affinity purification (LEHHHHHH) or an Avi tag for enzymatic biotinylation and 6-histidine tag (LEGLNDIFEAQKIEWHEGSHHHHHH). All proteins were expressed via isopropyl-β-d-thiogalactopyranoside (IPTG) induction (0.5 mM) in TBII media (MP Biomedicals, catalog no. 113046022-CF). Cells were pelleted, resuspended in high-salt buffer 1 [20 mM tris, 500 mM NaCl, and 20 mM imidazole (pH 8)], lysed via sonication, and centrifuged at high speed. Protein was purified from cleared lysates with metal affinity chromatography: 0.5 ml of Ni-NTA–agarose resin (QIAGEN, catalog no. 30210) was added per 1 liter of expression culture; resin was collected, washed, and eluted [20 mM tris, 150 mM NaCl, and 300 mM imidazole (pH 8)] over a 20-ml gravity column (Bio-Rad, catalog no. 7321010). Proteins were further purified via size exclusion chromatography (SEC) (Superdex 75 10/300 GL, GM), and fractions containing monomeric protein were collected and concentrated via centrifugal filtration with a 3-kDa molecular weight cutoff (Millipore). Avi-tagged proteins were enzymatically biotinylated in vitro with BirA (Avidity, catalog no. BirA500), followed by metal affinity purification or in vivo via coexpression of BirA (*E. coli* expression strain AVB101; Avidity, catalog no. CVB101). Aliquots of purified protein were snap-frozen in liquid nitrogen and stored at −80°C.

For use in crystallography experiments, designed proteins were expressed with a C-terminal extension including a sequence-specific nickel-assisted cleavage (SNAC) tag (*33*) followed by a 6-histidine tag. Designs were expressed and purified by immobilized metal affinity chromatography as above, dialyzed into SNAC buffer [100 mM acetone oxime, 100 mM CHES, and 100 mM NaCl (pH 8.6)], and the C-terminal tag cleaved by addition of 5 mM NiCl_2_ and incubation overnight at 4°C. A 10 mM EDTA was added to sequester residual nickel, and cleaved protein further purified by SEC (Superdex 75 as above). Recombinant BAK with deletion of 22 amino acids at the N terminus, deletion of 25 amino acids at the C terminus, and substitution of cysteine-166 to serine (BAK DN22DC25 C166S) was expressed in *E. coli* strain BL21 (DE3) with glutathione *S*-transferase (GST) fusion at the BAK N terminus as previously described (*8*, *23*). Bacterial cells were cultured at 37°C in super broth until an optical density of 1 was achieved at 600 nm, and cells were then induced for 3 hours with 1 mM IPTG. The resultant cells were harvested by centrifugation and lysed using a French press in a lysis buffer consisting of 50 mM tris (pH 8), 1 mM EDTA, 150 mM NaCl and deoxyribonuclease I (DNase I) (1.5 mg/ml; Roche, catalog no. 03724778103). The GST-BAK fusion protein was purified using glutathione resin (GenScript, catalog no. L00206). The GST fusion was removed by proteolysis using HRV-3C protease (1 ml at 0.2 mg/ml) overnight at 4°C, eluting BAK samples as the flowthrough from the column using lysis buffer without the DNase. BAK DN22DC25 C166S monomeric protein was purified by SEC using a Superdex 75 10/300 column in tris-buffered saline [TBS; 20 mM tris (pH 8) and 150 mM sodium chloride).

For liposome experiments, BAK DN22DC25 C166S modified with a C-terminal 6-histidine tag was expressed and purified as described above for BAK DN22DC25 C166S. FL-BAX (human BAX with C62S and C125S mutations) and BAXdC21 (as FL-BAX with the final 21 residues deleted and replaced with a 6-histidine tag) were cloned into a pTYB1 vector and expressed as C-terminal intein-chitin–binding fusions in bacterial cells at 37°C in super broth until an optical density of 1 was achieved at 600 nm, and cells were then induced for 3 hours with 1 mM IPTG. The resultant cells were harvested by centrifugation and lysed using a French press in a lysis buffer consisting of 20 mM tris (pH 8), 1 mM EDTA, 500 mM NaCl, and DNase I (1.5 mg/ml; Roche, catalog no. 03724778103). The BAX-intein-chitin–binding fusion proteins were purified from cell extracts by chitin affinity resin and on column intein self-cleavage with 50 mM dithiothreitol (DTT) over 2 days, followed by SEC using a Superdex 75 10/300 column in TBS [20 mM tris (pH 8) and 150 mM sodium chloride].

cBID was expressed as a GST fusion using the pGEX4T-1 vector in BL21 (DE3) *E. coli* cells. Bacterial cells were cultured at 37°C in super broth until an optical density of 0.6 was achieved at 600 nm, and cells were then induced for 16 hours with 1 mM IPTG at 18°C. The resultant cells were harvested by centrifugation and lysed using a French press in a lysis buffer consisting of 50 mM tris (pH 8), 1 mM EDTA, 150 mM NaCl, and DNase I (1.5 g/ml; Roche, catalog no. 03724778103). The cBID GST-fusion protein were purified using glutathione resin (GenScript, catalog no. L00206). The GST fusion was removed by proteolysis using thrombin protease (1 unit; Sigma-Aldrich, catalog no.10602400001) and adding CaCl_2_ to a final concentration of 1 mM, incubating overnight at 4°C, and eluting cBID as the flowthrough from the column.

### Affinity and specificity maturation by YSD

#### General methods

DNA libraries as described below or genes encoding initial computational designs were cloned into the pETCON plasmid (*34*), transformed into the *Saccharomyces cerevisiae* strain EBY100 by electroporation, and expressed with N-terminal fusion to Aga2p for surface display and C-terminal myc-tag for detection (*22*). Yeast libraries were grown in minimal media selective for the yeast strain (-ura) and the transforming plasmid (-trp), and protein expression was induced with 2% galactose. Surface expression was detected with anti–myc–fluorescence in situ hybridization (Immunology Consultants Laboratory, catalog no. RMYC-45F), and binding to biotinylated BAK or BAX proteins after co-incubation for 0.5 to 2 hours at 22°C was detected with phycoerythrin-streptavidin (Invitrogen/Thermo Fisher Scientific, catalog no. S866). Yeast were sorted with a SH800 (Sony) cell sorter, recovered to an optical density at 600 (OD_600_) > 1, and either pelleted for batch DNA extraction and deep sequencing (SSM libraries) or plated on solid media for isolating and sequencing individual clones (design screen and combinatorial libraries).

#### Library generation

SSM libraries were generated with overlap polymerase chain reaction (PCR) (*35*) using Phusion polymerase and custom degenerate primers to introduce mutations to NNK at each codon. Mutations with highest enrichment in the sorted SSM (fitness for the attribute promoted by the sort condition, either specific binding or high-affinity on-target binding) were combined in combinatorial libraries, generated by oligo assembly with primers having degenerate codons. Specificity and on-target affinity were independently assayed, and mutants independently enriched for both attributes were included in subsequent combinatorial libraries. The diversity of all combinatorial libraries was limited to less than 4 × 10^7^ variants.

#### Deep sequencing analysis

Yeast (2 × 1.0 OD units) were lysed with zymolase (125 U/ml) at 37°C for 5 hours, and DNA was harvested (Zymoprep kit from Zymo Research, catalog no. D2001). Genomic DNA was digested with exonuclease I (2 U/μl) and Lambda exonuclease (0.25 U/μl; New England Biolabs, catalog no. M0293S and M0262S) for 90 min at 30°C and plasmid DNA purified with a QIAquick kit (QIAGEN, catalog no. 28104). DNA was deep-sequenced with a MiSeq sequencer (Illumina): genes were PCR-amplified using primers that annealed to external regions within the plasmid, followed by a second round of PCR to add flanking sequences for annealing to the Illumina flow cell oligonucleotides and a 6–base pair sample identification sequence. High-fidelity Phusion polymerase was used for PCR, and round 1 was run for 12 cycles. PCR round 2 was monitored qualitatively with quantitative PCR (SYBR Safe), and cycles proceeded until amplification was exponential but was stopped before signal plateau. Barcodes were read on a MiSeq sequencer using either a 300-cycle or 600-cycle reagent kit (Illumina), and sequences were analyzed with custom scripts adapted from Enrich (*36*).

### Biophysical characterization

#### Biolayer interferometry

Kinetic and equilibrium binding properties of designed proteins versus all six prosurvival and both proapoptotic homologs were determined with BLI. Data were collected on an Octet RED96 (Forte Bio) and processed using the instrument’s integrated software. All proteins were diluted from concentrated stock in binding buffer [10 mM Hepes (pH 7.4), 150 mM NaCl, 3 mM EDTA, 0.05% surfactant P20, and 0.5% nonfat dry milk]. Streptavidin-coated biosensors were dipped in wells containing biotinylated BCL-2 proteins (25 nM) in binding buffer for 3 to 5 min for immobilization. After baseline measurement in buffer alone, binding kinetics were monitored by dipping the biosensors in wells containing defined concentrations of the designed protein (association) and then dipping sensors back into baseline wells (dissociation). Titrations were done in triplicate, and kinetic constants were determined from the mathematical fit of a 1:1 binding model.

#### Circular dichroism

Circular dichroism (CD) spectra were recorded with a J-1500 Circular Dichroism Spectrometer (JASCO), at a protein concentration of 10 μM in Dulbecco’s phosphate-buffered saline (DPBS) free of MgCl_2_ and NaCl (Life Technologies). Thermal denaturation was carried out from 25 to 95°C over 2 hours. GuHCl melt data were collected at 25°C.

### Crystal structure determination and refinement

#### Cocrystal structure of BAK bound αBAK2

BAK DN22DC25 C166S monomers were mixed in a 1:1.5 molar excess of the BAK binder (αBAK2) and then purified by SEC using a Superdex S200 10/300 column in TBS. The BAK DN22DC25 C166S monomer-αBAK2 complexes peaks were concentrated to 4 mg/ml. Crystals were obtained by the vapor diffusion method using a precipitant solution consisting 0.1 M MES (pH 6.5); 0.2 M l-proline; and 10% (w/v) polyethylene glycol molecular weight 3350 (PEG-3350). Crystals were cryoprotected by supplementing the precipitant solution with 15% (v/v) ethylene glycol before flash cooling in liquid nitrogen. X-ray diffraction data were collected at the Australian Synchrotron MX2 beamline at 13 keV using an Eiger 16 M detector (*37*). Data were indexed then integrated in x-ray detector software (XDS) (*38*) and scaled in AIMLESS (*39*). The phase problem was solved by molecular replacement with Phaser (*40*, *41*). The search model was constructed using the BCL-2-inhibitor complex as a reference model (PDB ID: 5JSN), aligning a BAK a3-a5 core (residues 90 to 142 from PDBID 2IMT) and a truncated model of the BAK binder (residues 7 to 33, 43 to 74, and 90 to 115), and the BAK and BAK binder models were merged for molecular replacement (*14*, *23*). The crystallographic models were iteratively refined using phenix.refine (*42*) and manually in real space with Coot (*43*). Images were rendered using Pymol 2.3.4 (Schrödinger, LLC).

#### Crystal structure of monomeric αBAK2

All crystallization trials were carried out at 20°C in 96-well format using the sitting-drop method. Crystal trays were set up using Mosquito by SPT Labtech. Drop volumes ranged from 200 to 400 nl and contained protein–to–crystallization solution in ratios of 1:1, 2:1, and 1:2. Diffraction quality crystals appeared in 0.1 M sodium Hepes (pH 7.5), 2% (v/v) PEG-400, and 2.0 M ammonium sulfate. Crystals were subsequently harvested in a cryo-loop and flash-frozen directly in liquid nitrogen without using any cryo-protectant for synchrotron data collection. Data collection from crystal of 02G10S1 was performed with synchrotron radiation at the Advanced Light Source 8.2.2. Crystals belonged to space group P41 21 2 with cell dimensions *a* = *b* = 53.829 Å and *c* = 206.038 Å, α = β = γ = 90°. X-ray intensities and data reduction were evaluated and integrated using XDS (*38*) and merged/scaled using Pointless/Aimless in the CCP4 program suite (*44*). Starting phases were obtained by molecular replacement using Phaser (*40*) using the designed model for the structures. Following molecular replacement, the models were improved using phenix.autobuild (*45*); efforts were made to reduce model bias by setting rebuild-in-place to false and using simulated annealing and prime-and-switch phasing. Structures were refined in Phenix (*45*). Model building was performed using Coot (*46*). The final model was evaluated using MolProbity (*47*). Data collection and refinement statistics are recorded in table S6.

Atomic coordinates and structure factors reported in this paper have been deposited in the PDB (www.rcsb.org/) with accession codes 9CLB (BAK:αBAK2 complex) and 8EJA (unbound αBAK2).

### In vitro mechanism assays

#### Generation of MEFs expressing human BAK

A retroviral expression construct in the pMIG vector (murine stem cell virus–internal ribosomal entry site–green fluorescent protein) expressing human BAK was transiently transfected into Phoenix ecotropic packaging cells using X-tremeGENE (Merck, catalog no. XTGHP-RO). Filtered virus-containing supernatant was then used to infect *BAX^−/−^BAK^−/−^* MEFs by spin inoculation as previously described (*10*). Cells stably expressing human BAK were then selected by FACS-sorting GFP^+^ cells. Cells were maintained in Dulbecco’s Modified Eagle’s KELSO formulation (DME KELSO) medium supplemented with 10% (v/v) fetal bovine serum, 250 mM l-asparagine, and 50 mM 2-mercaptoethanol.

#### Liposome release assay

Liposome assays were performed as has been described previously (*6*, *48*). Briefly, liposomes were prepared to entrap the self-quenching dye 5(6)-carboxy-fluorescein using a cocktail of lipids to mimic the composition of the MOM (46% phosphatidylcholine, 25% phosphatidylethanolamine, 11% phosphatidylinositol, 10% phosphatidylserine, and 8% cardiolipin) and supplemented with 5% of a nickel-chelating lipid (1,2-dioleoyl-*sn*-glycero-3-[*N*-(5-amino-1-carboxypentyl)iminodiacetic-acid)-succinyl]) for experiments using his-tagged BAK or BAX, which had no transmembrane domain (BAK DN22DC25 and BAX DC21). Purified protein samples were diluted in either TBS buffer and appropriate volumes added to a final volume of 150 μl containing liposomes (4 μg/ml) in a small unilamellar vesicle (SUV) buffer [10 mM Hepes (pH 7.5), 135 mM KCl, and 1 mM MgCl_2_]. For assays requiring cBid, the protein was expressed and purified as described previously (*6*, *49*) and added to a final concentration of 500 μM in the liposome assay. For assays with heat treatment, samples were heated at 43°C for 40 min and then incubated at room temperature with shaking for 15 min before fluoresce measurements described below. To fully permeabilized liposomes, CHAPS detergent [final concentration of 1% (w/v)] was added as a control for maximal fluorescence. Minimal fluorescence was determined with SUV buffer and liposomes alone. All experiments were performed in triplicate, and experiments were repeated independently three times. Release fractions were calculated as a percentage of maximal fluorescence compared to the CHAPS control. Assays were incubated at room temperature for up to 120 min, and fluorescence measurements were recorded every 2 min with excitation at 485 nm and emission at 535 nm. IC_50_ values for αBAK2, αBAK1, and CDP02 were determined using GraphPad Prism fitting to a [Inhibitor] versus response-variable slope (four parameters) equation, taking the mean IC_50_ value from three independent experiments.

To test if αBAK2 could disrupt BAK oligomers, blue native PAGE followed by Western blots was used. A liposome assay for BAK was performed as described above with 38 nM BAK incubated with 15 nM cBID for 30 min before adding αBAK2 at various concentrations (10 to 62 nM) for 30 min at room temperature. Samples were then treated with 50 mM EDTA before running a NuPAGE blue native gel (Thermo Fisher Scientific, catalog no. BN1002BOX) according to the standard protocols for Western blotting and transferred to a polyvinylidene difluoride membrane. Immunoblotting was performed BAK [1:2000; clone 7D10 (AG2), rat monoclonal] (*50*, *51*). Secondary detection used a horseradish peroxidase (HRP)–conjugated anti-rat (1:5000, #3010–05, Southern Biotech, RRID: AB_2795801) secondary antibody. Chemiluminescence detection was performed with Luminata Forte Western HRP substrate (Millipore, Cat #WBLUF0500) with a ChemiDoc XRS + System (Bio-Rad). Images processed with ImageLab Software (Bio-Rad).

#### *Cytochrome *c* release assays*

To test BAK function full-length wildtype human BAK was expressed in SV40 immortalized MEFs lacking endogenous mouse BAK and BAX (BAK^−/-^BAX^−/−^ MEF) (*52*). Cells were maintained at 37°C with 10% CO_2_ in a humidified incubator. Cells were cultured in Dulbecco’s Modified Eagle Medium (DMEM, Fisher Scientific, catalog no. 11–965-092) supplemented with penicillin, streptomycin, 10% (v/v) fetal calf serum (FCS), 0.1 mM L-asparagine and 55 μM 2-mercaptoethanol (2-ME). Cells were harvested by trypsin digestion, washed in PBS, collected by centrifugation at 600 g for 5 min, then resuspended at a concentration of 1x 10^7^ cells/ml (1x10^6^ in 100 μl) in sucrose buffer supplemented with digitonin (100 mM sucrose, 20 mM HEPES-NaOH pH 7.5, 100 mM KCl, 2.5 mM MgCl2, 4 mg/ml Pepstatin A (Sigma-Aldrich, catalog no. EI10), Complete protease inhibitors, EDTA-free (Roche, catalog no. COEDTAF-RO) and 0.025% w/v digitonin (Calbiochem, Merck, catalog no. 300410). Cells were incubated for 10 min on ice to permeabilize the cell membrane and maintain intact mitochondria. Membrane fractions were collected by centrifugation at 16,000 g for 5 min and the supernatant discarded. Pellets were resuspended in sucrose buffer (without digitonin) prior to incubation for 10 min at room temperature with αBAK2 inhibitor (ranging from 4 nM to 1 μM), followed by incubation for 30 min at 30°C with 20 nM cBID to activate the BAK protein. Following αBAK2 and cBID incubations, each sample was assayed for cytochrome *c* release (*53*).

To measure cytochrome *c* release from mitochondria following BAK/BAX-mediated pore formation, 25 μl samples were subject to centrifugation at 16,000 g for 5 min. When mitochondria were permeabilized cytochrome *c* was released from the membrane pellet fraction to the soluble supernatant fraction. Reducing SDS sample buffer was added to each fraction (25 μl to supernatants, 50 μl to pellets). Cytochrome *c* samples were boiled, and then resolved by SDS-PAGE (12% TGX gels, Bio-Rad, Cat #4561043) and transferred to nitrocellulose. Immunoblotting was performed for cytochrome *c* (1:2000, clone 7H8.2C12, mouse monoclonal, catalog number 556433, BD Biosciences, RRID: AB_396417). Secondary detection used a HRP-conjugated anti-mouse (1:5000; #1010-05, Southern Biotech, RRID: AB_2619742) secondary antibody. After blotting for cytochrome *c*, blots were reblotted for VDAC1 (1:5000; polyclonal rabbit, AB10527, Millipore, RRID: AB_10806766). Secondary detection used a HRP-conjugated anti-rabbit (1:5000; #4030-05, Southern Biotech, RRID: AB_2687483) secondary antibody. Chemiluminescence detection was performed as described above for BAK immunoblotting.

To test BAX function, mouse liver mitochondria (MLM) were prepared from wild-type or BAK^−/−^ C57BL/6 wild-type mice as described (*54*, *55*). This protocol removes all endogenous BAX. A 50 μl of the isolated mitochondria [1 mg/ml based on absorbance at 280 nm (*A*_280_)] were then treated with recombinant FL-BAX (final concentration of 10 nM), and appropriate concentrations of αBAX2 (final concentrations of 25 to 120 μM) and optionally cBID (final concentration of 20 nM) in MELB buffer [100 mM KCl, 2.5 mM MgCl_2_, 100 mM sucrose, 20 mM Hepes/KOH (pH 7.5), and 5 mM DTT]. Supernatant and pellet fractions were separated by centrifugation at 10,000*g* for 5 min. Reducing sample buffer was added to each fraction (16 μl of 4X buffer to supernatants and 66 μl of 1X buffer to pellets), boiled, and then resolved by SDS-PAGE (BoltTM bis-tris plus 4 to 12% gels, Invitrogen, catalog no. NW04120BOX) and transferred to nitrocellulose. Immunoblotting was performed for cytochrome *c* and VDAC1 as described above for MEF experiments.

## References

[1] A. Peña-Blanco, A. J. García-Sáez, Bax, Bak and beyond—Mitochondrial performance in apoptosis. FEBS J. 285, 416–431 (2018). 28755482 10.1111/febs.14186

[2] T. Moldoveanu, C. R. Grace, F. Llambi, A. Nourse, P. Fitzgerald, K. Gehring, R. W. Kriwacki, D. R. Green, BID-induced structural changes in BAK promote apoptosis. Nat. Struct. Mol. Biol. 20, 589–597 (2013). 23604079 10.1038/nsmb.2563 PMC3683554

[3] L. D. Walensky, K. Pitter, J. Morash, K. J. Oh, S. Barbuto, J. Fisher, E. Smith, G. L. Verdine, S. J. Korsmeyer, A stapled BID BH3 helix directly binds and activates BAX. Mol. Cell 24, 199–210 (2006). 17052454 10.1016/j.molcel.2006.08.020

[4] E. Gavathiotis, M. Suzuki, M. L. Davis, K. Pitter, G. H. Bird, S. G. Katz, H.-C. Tu, H. Kim, E. H.-Y. Cheng, N. Tjandra, L. D. Walensky, BAX activation is initiated at a novel interaction site. Nature 455, 1076–1081 (2008). 18948948 10.1038/nature07396 PMC2597110

[5] M. Suzuki, R. J. Youle, N. Tjandra, Structure of Bax: Coregulation of dimer formation and intracellular localization. Cell 103, 645–654 (2000). 11106734 10.1016/s0092-8674(00)00167-7

[6] J. M. Brouwer, P. Lan, A. D. Cowan, J. P. Bernardini, R. W. Birkinshaw, M. F. van Delft, B. E. Sleebs, A. Y. Robin, A. Wardak, I. K. Tan, B. Reljic, E. F. Lee, W. D. Fairlie, M. J. Call, B. J. Smith, G. Dewson, G. Lessene, P. M. Colman, P. E. Czabotar, Conversion of Bim-BH3 from activator to inhibitor of Bak through structure-based design. Mol. Cell 68, 659–672.e9 (2017). 29149594 10.1016/j.molcel.2017.11.001

[7] P. E. Czabotar, D. Westphal, G. Dewson, S. Ma, C. Hockings, W. D. Fairlie, E. F. Lee, S. Yao, A. Y. Robin, B. J. Smith, D. C. S. Huang, R. M. Kluck, J. M. Adams, P. M. Colman, Bax crystal structures reveal how BH3 domains activate Bax and nucleate its oligomerization to induce apoptosis. Cell 152, 519–531 (2013). 23374347 10.1016/j.cell.2012.12.031

[8] J. M. Brouwer, D. Westphal, G. Dewson, A. Y. Robin, R. T. Uren, R. Bartolo, G. V. Thompson, P. M. Colman, R. M. Kluck, P. E. Czabotar, Bak core and latch domains separate during activation, and freed core domains form symmetric homodimers. Mol. Cell 55, 938–946 (2014). 25175025 10.1016/j.molcel.2014.07.016

[9] A. D. Cowan, N. A. Smith, J. J. Sandow, E. A. Kapp, Y. H. Rustam, J. M. Murphy, J. M. Brouwer, J. P. Bernardini, M. J. Roy, A. Z. Wardak, I. K. Tan, A. I. Webb, J. M. Gulbis, B. J. Smith, G. E. Reid, G. Dewson, P. M. Colman, P. E. Czabotar, BAK core dimers bind lipids and can be bridged by them. Nat. Struct. Mol. Biol. 27, 1024–1031 (2020). 32929280 10.1038/s41594-020-0494-5

[10] E. F. Lee, P. E. Czabotar, M. F. van Delft, E. M. Michalak, M. J. Boyle, S. N. Willis, H. Puthalakath, P. Bouillet, P. M. Colman, D. C. S. Huang, W. D. Fairlie, A novel BH3 ligand that selectively targets Mcl-1 reveals that apoptosis can proceed without Mcl-1 degradation. J. Cell Biol. 180, 341–355 (2008). 18209102 10.1083/jcb.200708096 PMC2213596

[11] A. J. Souers, J. D. Leverson, E. R. Boghaert, S. L. Ackler, N. D. Catron, J. Chen, B. D. Dayton, H. Ding, S. H. Enschede, W. J. Fairbrother, D. C. S. Huang, S. G. Hymowitz, S. Jin, S. L. Khaw, P. J. Kovar, L. T. Lam, J. Lee, H. L. Maecker, K. C. Marsh, K. D. Mason, M. J. Mitten, P. M. Nimmer, A. Oleksijew, C. H. Park, C.-M. Park, D. C. Phillips, A. W. Roberts, D. Sampath, J. F. Seymour, M. L. Smith, G. M. Sullivan, S. K. Tahir, C. Tse, M. D. Wendt, Y. Xiao, J. C. Xue, H. Zhang, R. A. Humerickhouse, S. H. Rosenberg, S. W. Elmore, ABT-199, a potent and selective BCL-2 inhibitor, achieves antitumor activity while sparing platelets. Nat. Med. 19, 202–208 (2013). 23291630 10.1038/nm.3048

[12] J. D. Leverson, H. Zhang, J. Chen, S. K. Tahir, D. C. Phillips, J. Xue, P. Nimmer, S. Jin, M. Smith, Y. Xiao, P. Kovar, A. Tanaka, M. Bruncko, G. S. Sheppard, L. Wang, S. Gierke, L. Kategaya, D. J. Anderson, C. Wong, J. Eastham-Anderson, M. J. C. Ludlam, D. Sampath, W. J. Fairbrother, I. Wertz, S. H. Rosenberg, C. Tse, S. W. Elmore, A. J. Souers, Potent and selective small-molecule MCL-1 inhibitors demonstrate on-target cancer cell killing activity as single agents and in combination with ABT-263 (navitoclax). Cell Death Dis. 6, e1590 (2015). 25590800 10.1038/cddis.2014.561 PMC4669759

[13] S. Dutta, J. Ryan, T. S. Chen, C. Kougentakis, A. Letai, A. E. Keating, Potent and specific peptide inhibitors of human pro-survival protein Bcl-x_L_. J. Mol. Biol. 427, 1241–1253 (2015). 25451027 10.1016/j.jmb.2014.09.030 PMC4357494

[14] S. Berger, E. Procko, D. Margineantu, E. F. Lee, B. W. Shen, A. Zelter, D.-A. Silva, K. Chawla, M. J. Herold, J.-M. Garnier, R. Johnson, M. J. MacCoss, G. Lessene, T. N. Davis, P. S. Stayton, B. L. Stoddard, W. D. Fairlie, D. M. Hockenbery, D. Baker, Computationally designed high specificity inhibitors delineate the roles of BCL2 family proteins in cancer. eLife 5, e20352 (2016). 27805565 10.7554/eLife.20352 PMC5127641

[15] R. M. Guerra, G. H. Bird, E. P. Harvey, N. V. Dharia, K. J. Korshavn, M. S. Prew, K. Stegmaier, L. D. Walensky, Precision targeting of BFL-1/A1 and an ATM Co-dependency in human cancer. Cell Rep. 24, 3393–3403.e5 (2018). 30257201 10.1016/j.celrep.2018.08.089 PMC6365304

[16] L. Wang, G. A. Doherty, A. S. Judd, Z.-F. Tao, T. M. Hansen, R. R. Frey, X. Song, M. Bruncko, A. R. Kunzer, X. Wang, M. D. Wendt, J. A. Flygare, N. D. Catron, R. A. Judge, C. H. Park, S. Shekhar, D. C. Phillips, P. Nimmer, M. L. Smith, S. K. Tahir, Y. Xiao, J. Xue, H. Zhang, P. N. Le, M. J. Mitten, E. R. Boghaert, W. Gao, P. Kovar, E. F. Choo, D. Diaz, W. J. Fairbrother, S. W. Elmore, D. Sampath, J. D. Leverson, A. J. Souers, Discovery of A-1331852, a first-in-class, potent, and orally-bioavailable BCL-X_L_ inhibitor. ACS Med. Chem. Lett. 11, 1829–1836 (2020). 33062160 10.1021/acsmedchemlett.9b00568 PMC7549103

[17] J. P. Pogmore, D. Uehling, D. W. Andrews, Pharmacological targeting of executioner proteins: Controlling life and death. J. Med. Chem. 64, 5276–5290 (2021). 33939407 10.1021/acs.jmedchem.0c02200

[18] J. Kale, E. J. Osterlund, D. W. Andrews, BCL-2 family proteins: Changing partners in the dance towards death. Cell Death Differ. 25, 65–80 (2018). 29149100 10.1038/cdd.2017.186 PMC5729540

[19] L. A. Barclay, T. E. Wales, T. P. Garner, F. Wachter, S. Lee, R. M. Guerra, M. L. Stewart, C. R. Braun, G. H. Bird, E. Gavathiotis, J. R. Engen, L. D. Walensky, Inhibition of Pro-apoptotic BAX by a noncanonical interaction mechanism. Mol. Cell 57, 873–886 (2015). 25684204 10.1016/j.molcel.2015.01.014 PMC4384643

[20] M. W. McHenry, P. Shi, C. M. Camara, D. T. Cohen, T. J. Rettenmaier, U. Adhikary, M. A. Gygi, K. Yang, S. P. Gygi, T. E. Wales, J. R. Engen, J. A. Wells, L. D. Walensky, Covalent inhibition of pro-apoptotic BAX. Nat. Chem. Biol. 20, 1022–1032 (2024). 38233584 10.1038/s41589-023-01537-6 PMC11252247

[21] E. Procko, G. Y. Berguig, B. W. Shen, Y. Song, S. Frayo, A. J. Convertine, D. Margineantu, G. Booth, B. E. Correia, Y. Cheng, W. R. Schief, D. M. Hockenbery, O. W. Press, B. L. Stoddard, P. S. Stayton, D. Baker, A computationally designed inhibitor of an Epstein-Barr viral Bcl-2 protein induces apoptosis in infected cells. Cell 157, 1644–1656 (2014). 24949974 10.1016/j.cell.2014.04.034 PMC4079535

[22] G. Chao, W. L. Lau, B. J. Hackel, S. L. Sazinsky, S. M. Lippow, K. D. Wittrup, Isolating and engineering human antibodies using yeast surface display. Nat. Protoc. 1, 755–768 (2006). 17406305 10.1038/nprot.2006.94

[23] T. Moldoveanu, Q. Liu, A. Tocilj, M. Watson, G. Shore, K. Gehring, The X-ray structure of a BAK homodimer reveals an inhibitory zinc binding site. Mol. Cell 24, 677–688 (2006). 17157251 10.1016/j.molcel.2006.10.014

[24] R. W. Birkinshaw, S. Iyer, D. Lio, C. S. Luo, J. M. Brouwer, M. S. Miller, A. Y. Robin, R. T. Uren, G. Dewson, R. M. Kluck, P. M. Colman, P. E. Czabotar, Structure of detergent-activated BAK dimers derived from the inert monomer. Mol. Cell 81, 2123–2134.e5 (2021). 33794146 10.1016/j.molcel.2021.03.014

[25] C. Hockings, A. E. Alsop, S. C. Fennell, E. F. Lee, W. D. Fairlie, G. Dewson, R. M. Kluck, Mcl-1 and Bcl-x_L_ sequestration of Bak confers differential resistance to BH3-only proteins. Cell Death Differ. 25, 721–734 (2018). 29459767 10.1038/s41418-017-0010-6 PMC5864222

[26] F. Llambi, T. Moldoveanu, S. W. G. Tait, L. Bouchier-Hayes, J. Temirov, L. L. McCormick, C. P. Dillon, D. R. Green, A unified model of mammalian BCL-2 protein family interactions at the mitochondria. Mol. Cell 44, 517–531 (2011). 22036586 10.1016/j.molcel.2011.10.001 PMC3221787

[27] S. Yuan, R. Sun, H. Shi, N. M. Chapman, H. Hu, C. Guy, S. Rankin, A. Kc, G. Palacios, X. Meng, X. Sun, P. Zhou, X. Yang, S. Gottschalk, H. Chi, VDAC2 loss elicits tumour destruction and inflammation for cancer therapy. Nature 640, 1062–1071 (2025). 40108474 10.1038/s41586-025-08732-6 PMC12018455

[28] J. E. Chipuk, L. Bouchier-Hayes, D. R. Green, Mitochondrial outer membrane permeabilization during apoptosis: The innocent bystander scenario. Cell Death Differ. 13, 1396–1402 (2006). 16710362 10.1038/sj.cdd.4401963

[29] J. Karch, J. Q. Kwong, A. R. Burr, M. A. Sargent, J. W. Elrod, P. M. Peixoto, S. Martinez-Caballero, H. Osinska, E. H.-Y. Cheng, J. Robbins, K. W. Kinnally, J. D. Molkentin, Bax and Bak function as the outer membrane component of the mitochondrial permeability pore in regulating necrotic cell death in mice. eLife 2, e00772 (2013). 23991283 10.7554/eLife.00772 PMC3755340

[30] S. J. Fleishman, A. Leaver-Fay, J. E. Corn, E.-M. Strauch, S. D. Khare, N. Koga, J. Ashworth, P. Murphy, F. Richter, G. Lemmon, J. Meiler, D. Baker, RosettaScripts: A scripting language interface to the Rosetta macromolecular modeling suite. PLOS ONE 6, e20161 (2011). 21731610 10.1371/journal.pone.0020161 PMC3123292

[31] A. Leaver-Fay, M. Tyka, S. M. Lewis, O. F. Lange, J. Thompson, R. Jacak, K. W. Kaufman, P. D. Renfrew, C. A. Smith, W. Sheffler, I. W. Davis, S. Cooper, A. Treuille, D. J. Mandell, F. Richter, Y.-E. A. Ban, S. J. Fleishman, J. E. Corn, D. E. Kim, S. Lyskov, M. Berrondo, S. Mentzer, Z. Popović, J. J. Havranek, J. Karanicolas, R. Das, J. Meiler, T. Kortemme, J. J. Gray, B. Kuhlman, D. Baker, P. Bradley, Chapter 19 - Rosetta3: An object-oriented software suite for the simulation and design of macromolecules, in *Methods in Enzymology*, M. L. Johnson, L. Brand, Eds. (Academic Press, 2011), vol. 487, pp. 545–574. 10.1016/B978-0-12-381270-4.00019-6 PMC4083816 21187238

[32] Y. Zhang, J. Skolnick, TM-align: A protein structure alignment algorithm based on the TM-score. Nucleic Acids Res. 33, 2302–2309 (2005). 15849316 10.1093/nar/gki524 PMC1084323

[33] B. Dang, M. Mravic, H. Hu, N. Schmidt, B. Mensa, W. F. DeGrado, SNAC-tag for sequence-specific chemical protein cleavage. Nat. Methods 16, 319–322 (2019). 30923372 10.1038/s41592-019-0357-3 PMC6443254

[34] S. J. Fleishman, T. A. Whitehead, D. C. Ekiert, C. Dreyfus, J. E. Corn, E.-M. Strauch, I. A. Wilson, D. Baker, Computational design of proteins targeting the conserved stem region of influenza hemagglutinin. Science 332, 816–821 (2011). 21566186 10.1126/science.1202617 PMC3164876

[35] E. Procko, R. Hedman, K. Hamilton, J. Seetharaman, S. J. Fleishman, M. Su, J. Aramini, G. Kornhaber, J. F. Hunt, L. Tong, G. T. Montelione, D. Baker, Computational design of a protein-based enzyme inhibitor. J. Mol. Biol. 425, 3563–3575 (2013). 23827138 10.1016/j.jmb.2013.06.035 PMC3818146

[36] D. M. Fowler, C. L. Araya, W. Gerard, S. Fields, Enrich: Software for analysis of protein function by enrichment and depletion of variants. Bioinformatics 27, 3430–3431 (2011). 22006916 10.1093/bioinformatics/btr577 PMC3232369

[37] D. Aragão, J. Aishima, H. Cherukuvada, R. Clarken, M. Clift, N. P. Cowieson, D. J. Ericsson, C. L. Gee, S. Macedo, N. Mudie, S. Panjikar, J. R. Price, A. Riboldi-Tunnicliffe, R. Rostan, R. Williamson, T. T. Caradoc-Davies, MX2: A high-flux undulator microfocus beamline serving both the chemical and macromolecular crystallography communities at the Australian Synchrotron. J. Synchrotron Radiat. 25, 885–891 (2018). 29714201 10.1107/S1600577518003120 PMC5929359

[38] W. Kabsch, XDS. Acta Crystallogr. D Biol. Crystallogr. 66, 125–132 (2010). 20124692 10.1107/S0907444909047337 PMC2815665

[39] P. R. Evans, G. N. Murshudov, How good are my data and what is the resolution? Acta Crystallogr. D Biol. Crystallogr. 69, 1204–1214 (2013). 23793146 10.1107/S0907444913000061 PMC3689523

[40] A. J. McCoy, R. W. Grosse-Kunstleve, P. D. Adams, M. D. Winn, L. C. Storoni, R. J. Read, Phaser crystallographic software. J. Appl. Cryst. 40, 658–674 (2007). 19461840 10.1107/S0021889807021206 PMC2483472

[41] A. J. McCoy, Solving structures of protein complexes by molecular replacement with Phaser. Acta Crystallogr. D Biol. Crystallogr. 63, 32–41 (2007). 17164524 10.1107/S0907444906045975 PMC2483468

[42] P. V. Afonine, R. W. Grosse-Kunstleve, N. Echols, J. J. Headd, N. W. Moriarty, M. Mustyakimov, T. C. Terwilliger, A. Urzhumtsev, P. H. Zwart, P. D. Adams, Towards automated crystallographic structure refinement with *phenix.refine*. Acta Crystallogr. D Biol. Crystallogr. 68, 352–367 (2012). 22505256 10.1107/S0907444912001308 PMC3322595

[43] P. Emsley, B. Lohkamp, W. G. Scott, K. Cowtan, Features and development of *Coot*. Acta Crystallogr. D Biol. Crystallogr. 66, 486–501 (2010). 20383002 10.1107/S0907444910007493 PMC2852313

[44] M. D. Winn, C. C. Ballard, K. D. Cowtan, E. J. Dodson, P. Emsley, P. R. Evans, R. M. Keegan, E. B. Krissinel, A. G. W. Leslie, A. McCoy, S. J. McNicholas, G. N. Murshudov, N. S. Pannu, E. A. Potterton, H. R. Powell, R. J. Read, A. Vagin, K. S. Wilson, Overview of the CCP4 suite and current developments. Acta Crystallogr. D Biol. Crystallogr. 67, 235–242 (2011). 21460441 10.1107/S0907444910045749 PMC3069738

[45] P. D. Adams, P. V. Afonine, G. Bunkóczi, V. B. Chen, I. W. Davis, N. Echols, J. J. Headd, L.-W. Hung, G. J. Kapral, R. W. Grosse-Kunstleve, A. J. McCoy, N. W. Moriarty, R. Oeffner, R. J. Read, D. C. Richardson, J. S. Richardson, T. C. Terwilliger, P. H. Zwart, PHENIX: A comprehensive Python-based system for macromolecular structure solution. Acta Crystallogr. D Biol. Crystallogr. 66, 213–221 (2010). 20124702 10.1107/S0907444909052925 PMC2815670

[46] P. Emsley, K. Cowtan, *Coot*: Model-building tools for molecular graphics. Acta Crystallogr. D Biol. Crystallogr. 60, 2126–2132 (2004). 15572765 10.1107/S0907444904019158

[47] C. J. Williams, J. J. Headd, N. W. Moriarty, M. G. Prisant, L. L. Videau, L. N. Deis, V. Verma, D. A. Keedy, B. J. Hintze, V. B. Chen, S. Jain, S. M. Lewis, W. B. Arendall III, J. Snoeyink, P. D. Adams, S. C. Lovell, J. S. Richardson, D. C. Richardson, MolProbity: More and better reference data for improved all-atom structure validation. Protein Sci. 27, 293–315 (2018). 29067766 10.1002/pro.3330 PMC5734394

[48] L. J. Pagliari, T. Kuwana, C. Bonzon, D. D. Newmeyer, S. Tu, H. M. Beere, D. R. Green, The multidomain proapoptotic molecules Bax and Bak are directly activated by heat. Proc. Natl. Acad. Sci. U.S.A. 102, 17975–17980 (2005). 16330765 10.1073/pnas.0506712102 PMC1312392

[49] R. M. Kluck, M. D. Esposti, G. Perkins, C. Renken, T. Kuwana, E. Bossy-Wetzel, M. Goldberg, T. Allen, M. J. Barber, D. R. Green, D. D. Newmeyer, The pro-apoptotic proteins, Bid and Bax, cause a limited permeabilization of the mitochondrial outer membrane that is enhanced by cytosol. J. Cell Biol. 147, 809–822 (1999). 10562282 10.1083/jcb.147.4.809 PMC2156156

[50] G. Dewson, T. Kratina, H. W. Sim, H. Puthalakath, J. M. Adams, P. M. Colman, R. M. Kluck, To trigger apoptosis, Bak exposes its BH3 domain and homodimerizes via BH3:groove interactions. Mol. Cell 30, 369–380 (2008). 18471982 10.1016/j.molcel.2008.04.005

[51] G. Dewson, T. Kratina, P. Czabotar, C. L. Day, J. M. Adams, R. M. Kluck, Bak activation for apoptosis involves oligomerization of dimers via their α6 helices. Mol. Cell 36, 696–703 (2009). 19941828 10.1016/j.molcel.2009.11.008

[52] M. C. Wei, W. X. Zong, E. H. Cheng, T. Lindsten, V. Panoutsakopoulou, A. J. Ross, K. A. Roth, G. R. MacGregor, C. B. Thompson, S. J. Korsmeyer, Proapoptotic BAX and BAK: A requisite gateway to mitochondrial dysfunction and death. Science 292, 727–730 (2001). 11326099 10.1126/science.1059108 PMC3049805

[53] S. Iyer, R. T. Uren, R. M. Kluck, Probing BAK and BAX activation and pore assembly with cytochrome c release, limited proteolysis, and oxidant-induced linkage, in *BCL-2 Family Proteins: Methods and Protocols*, E. Gavathiotis, Ed. (Springer, 2019), pp. 201–216. 10.1007/978-1-4939-8861-7_14 30536008

[54] T. Lindsten, A. J. Ross, A. King, W. X. Zong, J. C. Rathmell, H. A. Shiels, E. Ulrich, K. G. Waymire, P. Mahar, K. Frauwirth, Y. Chen, M. Wei, V. M. Eng, D. M. Adelman, M. C. Simon, A. Ma, J. A. Golden, G. Evan, S. J. Korsmeyer, G. R. MacGregor, C. B. Thompson, The combined functions of proapoptotic Bcl-2 family members bak and bax are essential for normal development of multiple tissues. Mol. Cell 6, 1389–1399 (2000). 11163212 10.1016/s1097-2765(00)00136-2 PMC3057227

[55] R. T. Uren, G. Dewson, C. Bonzon, T. Lithgow, D. D. Newmeyer, R. M. Kluck, Mitochondrial release of pro-apoptotic proteins: electrostatic interactions can hold cytochrome *c* but not Smac/diablo to mitochondrial membraneS. J. Biol. Chem. 280, 2266–2274 (2005). 15537572 10.1074/jbc.M411106200

