# Supplementary material for: Computational design of potent and selective binders of BAK and BAX
Source: Sci Adv. 2025 Sep 5;11(36):eadt4170. doi: 10.1126/sciadv.adt4170 (PMC12412652; doi:10.1126/sciadv.adt4170)
Supplement: Supplementary file 1 — Figs. S1 to S12 Tables S1 to S6 [file sciadv.adt4170_sm.pdf]

Science Advances
AAAS

Supplementary Materials for

Computational design of potent and selective binders of BAK and BAX

Stephanie Berger *et al.*

Corresponding author: Richard W. Birkinshaw, birkinshaw.r@wehi.edu.au; David Baker, dabaker@uw.edu; Peter E. Czabotar, czabotar@wehi.edu.au

Sci. Adv. **11**, eadt4170 (2025)
DOI: 10.1126/sciadv.adt4170

This PDF file includes:

Figs. S1 to S12
Tables S1 to S6

Fig. S1.

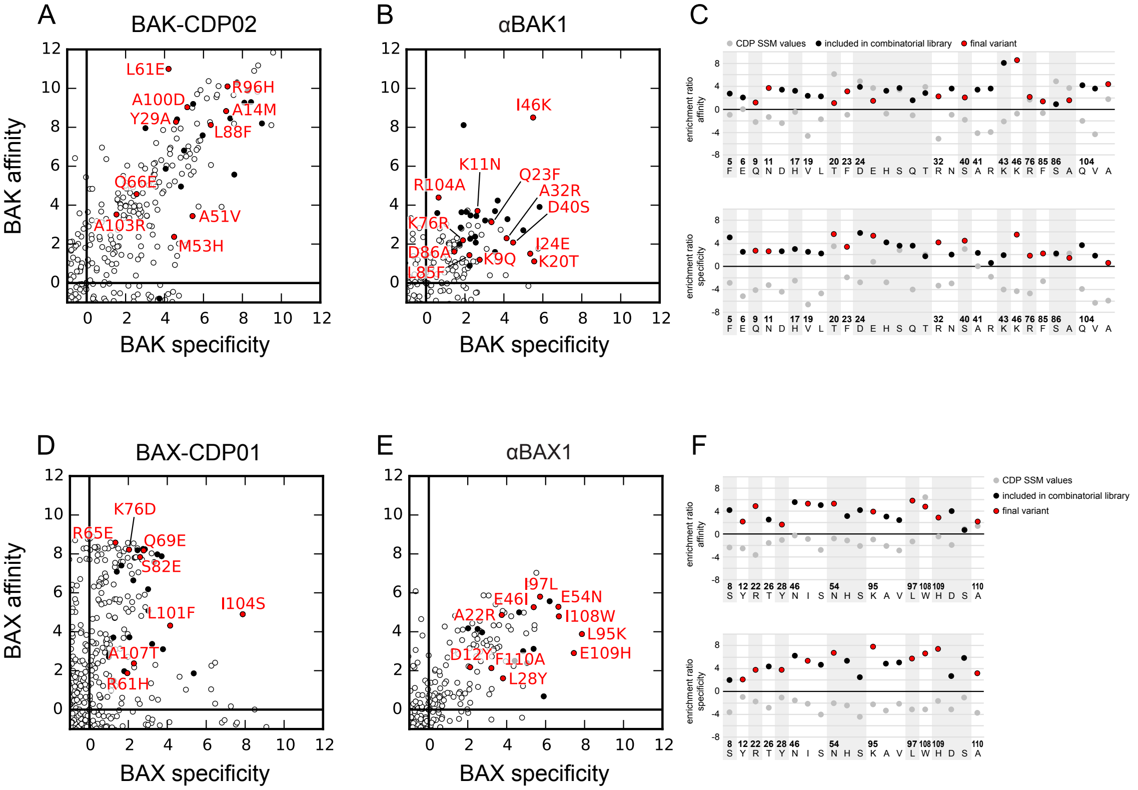

Selection of combinatorial mutants informed by SSM NGS data. Deep sequencing analysis of naïve and sorted SSM libraries, based on original CDPs (A,D) and the best variants from the first round of directed evolution, αBAK1 and αBAX1 (B,E), enabled quantitative analysis of the fitness of each single amino acid substitution for specificity or affinity toward BAK or BAX. (C, F) Enrichment ratios of mutations selected from αBAK1 and αBAX1 SSMs for inclusion in the second-generation combinatorial libraries were compared to enrichment ratios of the same mutations relative to the original CDPs (values from the first generation SSM); mutations enriched in the αBAK1 and αBAX1 SSMs were largely depleted in the CDP SSMs, and thus are likely context-dependent.

Fig. S2.

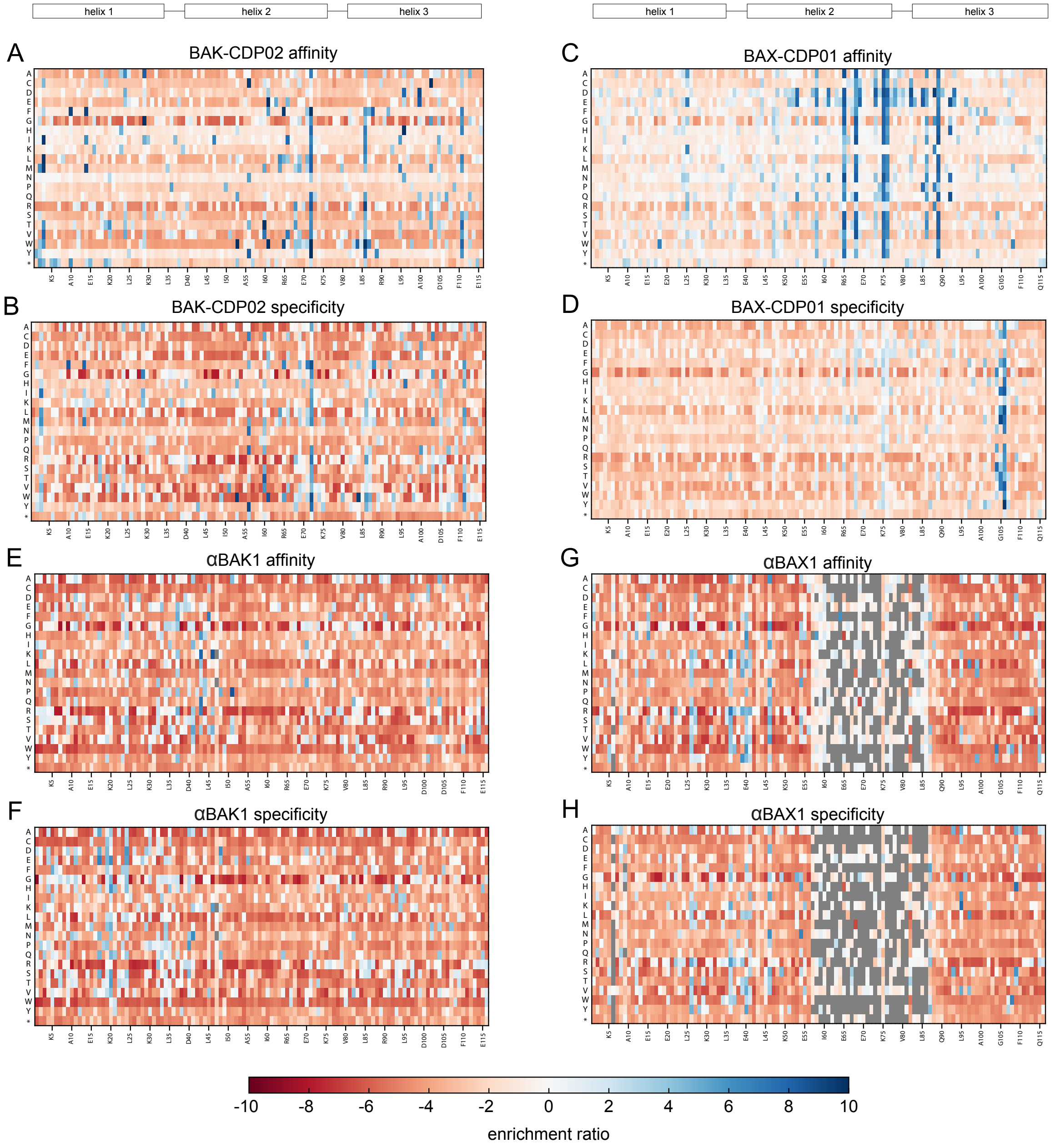

SSM heatmaps. SSM libraries based on BAK-CDP02 (A, B), BAX-CDP01 (C, D), αBAK1 (E, F) and αBAX1 (G, H) were screened for binding to labeled target homolog alone or in the presence of unlabeled pro-survival competitors. FACS-sorted pools were analyzed with NGS, and enrichment ratios were calculated as in Equation 1 (Materials and Methods). Enrichment or depletion of each mutant represents its fitness for high-affinity (A, C, E, G) or specific binding (B, D, F, H). Gray indicates positions where NGS data were poor quality and thus omitted from analysis.

Fig. S3.

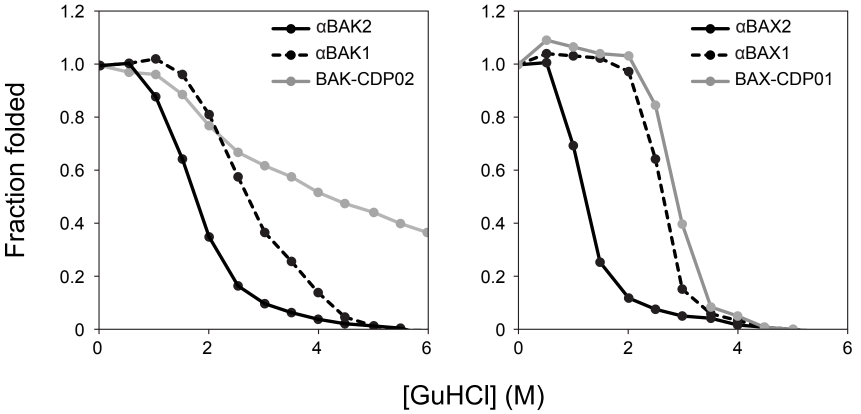

Designed protein characterization. Computationally designed proteins (gray) and their optimized successors (αBAK1 and αBAX1 dashed; αBAK2 and αBAX2 solid black) were denatured with guanidinium hydrochloride. CD signal at 222 nM was measured and loss of signal used to calculate the fraction folded. Values reported are the result of three accumulations averaged at acquisition.

Fig. S4.

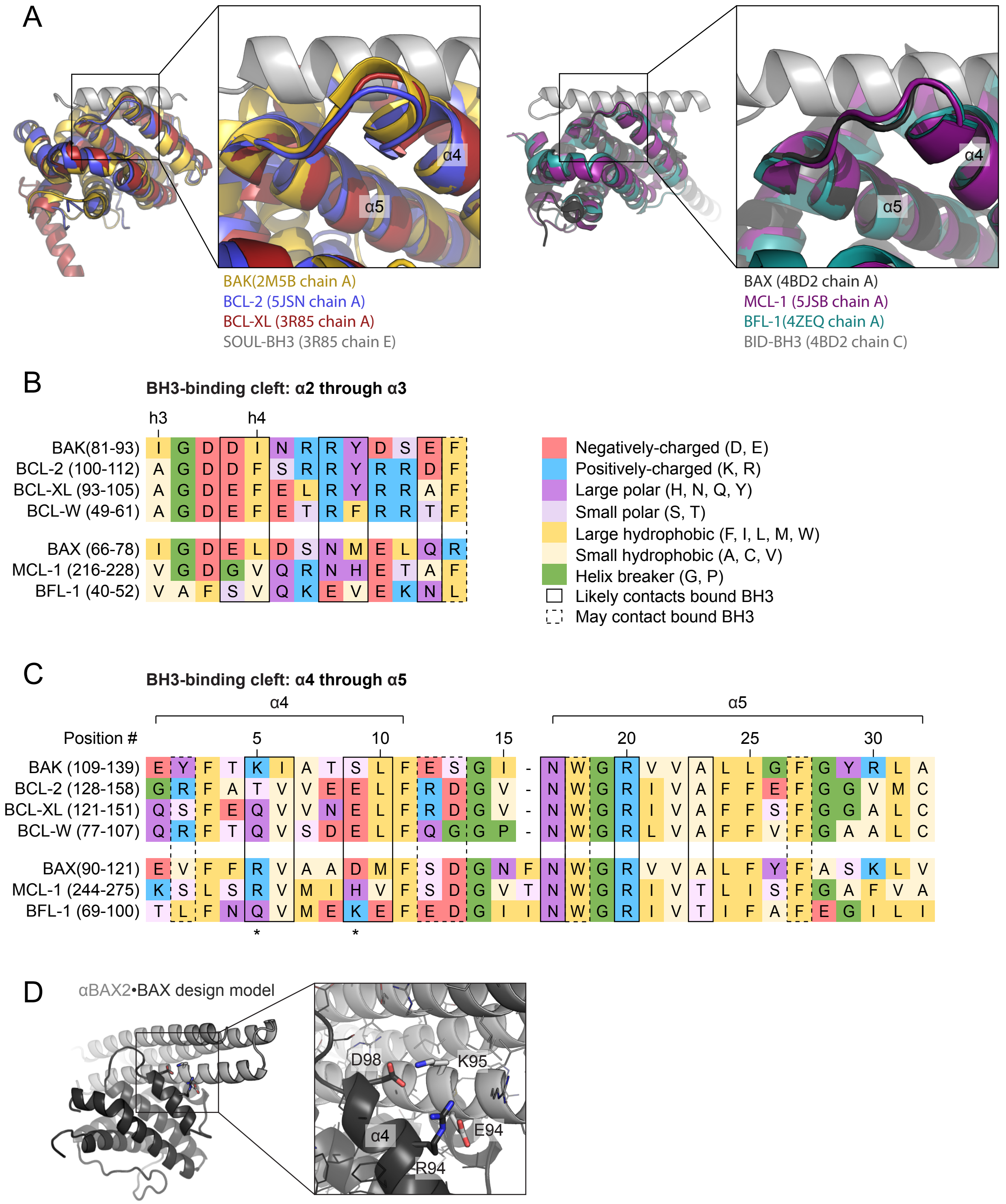

Structural basis for specificity profile of BAK- and BAX-targeting designed proteins. (A) Comparison of α4 and α5 of helix-bound models homologs of BCL-2 (5JSNa), BCL-XL (3R85a), and BAK (2M5B; left, bound to representative helix SOUL-BH3 [3R85e]) with MCL-1 (5JSBa), BFL-1 (4ZEQa) and BAX (4BD2a; right, bound to representative helix BID-BH3

[4BD2c]). While helices α4 and α5 are structurally similar, the loop between them is one residue shorter for BCL-2, BCL-XL and BAK than MCL- 1, BFL-1 and BAX, perhaps contributing to αBAK1 and αBAK2's cross-reactivity with BCL-2, and αBAK1 and αBAX2's cross-reactivity with BFL-1. (B,C) The denoted sequences of each homolog are aligned and colored by the indicated residue type. Black boxes indicate positions that would likely contact a bound helix, and dashed boxes indicate positions that may contact a bound helix depending on its side chain identity. (B) Structural evidence of the unique cross-reactivities is found on the opposite side of the BH3-binding cleft at positions spanning α2 and α3, where BAK is most similar to BCL-2 and BAX is most similar to MCL-1 and BFL-1, especially at interface positions. Annotations h3 and h4 indicate the hydrophobic residue positions that are characteristic of the BH3 motif. (C) The sequence of α5 is highly conserved among homologs, likely because it is buried in the core of the protein. However, α4 has considerable sequence diversity at interface positions, and thus the designed inhibitors likely take advantage of this region to gain specificity. (D) For example, αBAX2 residue K95 complements BAX residue D98 and αBAX2 E94 complements BAX R94; no other homolog has this exact electrostatic profile.

Fig. S5.

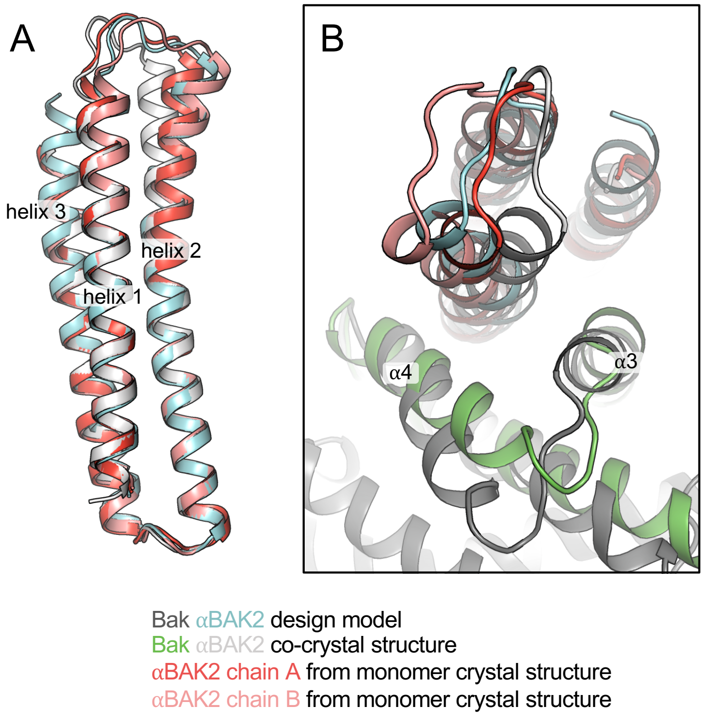

Crystal structure of unbound αBAK2. (A) Alignment of the two unbound αBAK2 chains in the asymmetric unit with the BAK-bound αBAK2 design model and αBAK2 monomer from the BAK:αBAK2 co-crystal structure. The αBAK2 design model (cyan) and the two chains from the unbound crystal structure (red and pink) deviate significantly from the BAK-bound crystal structure of αBAK2 (white) at the N-terminal end of helix 2 (B) Alignment of αBAK2 design model and crystal structures in context of BAK. The BAK α3-α4 loop is significantly different in the design model compared to the cocrystal structure. In the design model, αBAK2 sits closer to BAK α4 which is in a more open conformation than the crystal structure.

Fig. S6.

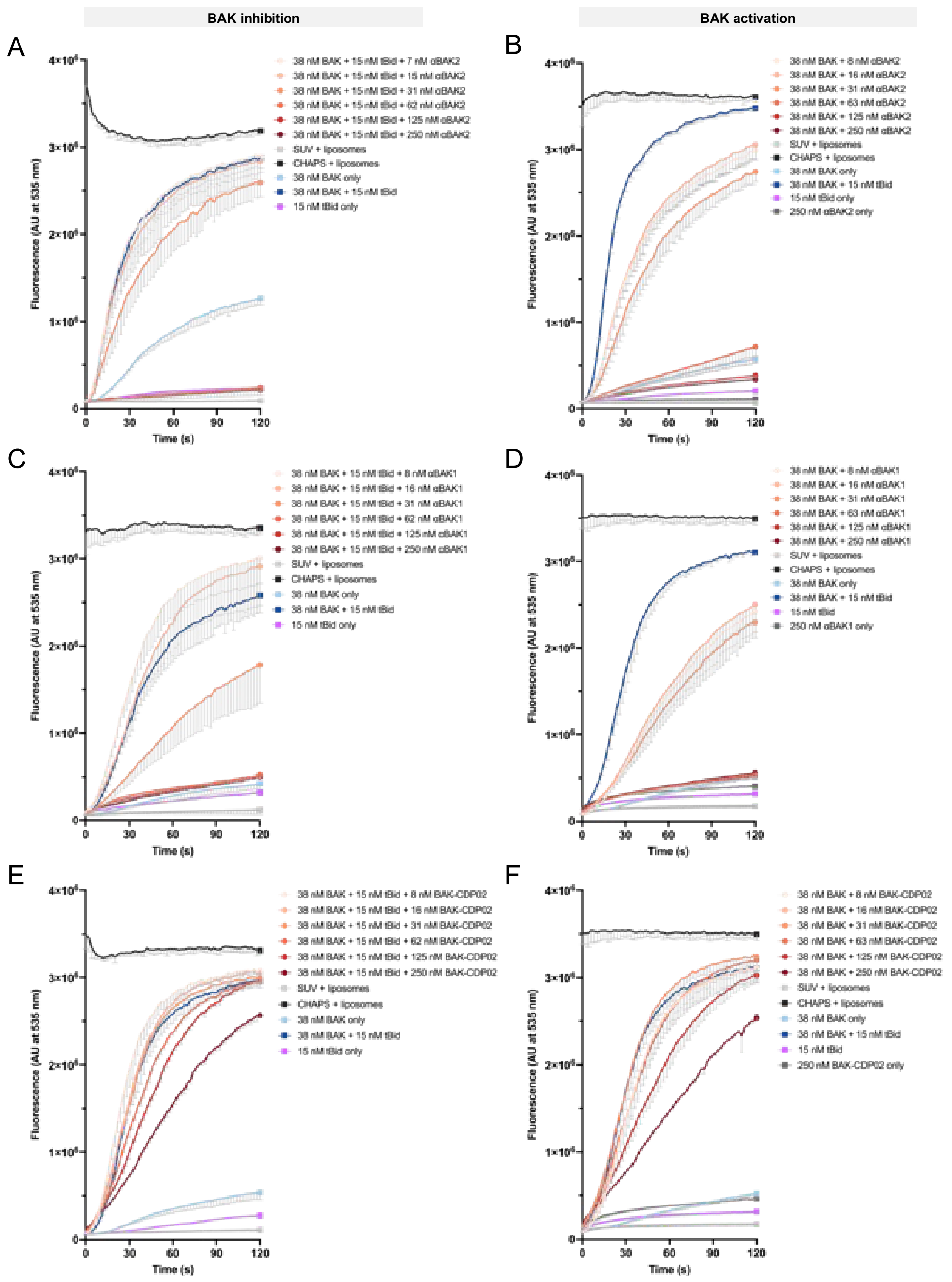

Time course showing BAK binders activate or inhibit BAK, depending on binding affinity and relative concentration. (A, C, E) Liposomes were treated with BAK, an activating concentration of BOP cBID, and a titration of each binder to determine their propensity to inhibit BAK-mediated permeabilization, or (B, D, F) treated with BAK and a titration of binder to determine their propensity to activate BAK-mediated permeabilization. Fluorescence intensity of the assay solution, as a proxy for liposome permeabilization, was monitored over time (min). Data a represented in Figure 3 as the endpoint of this assay. Data shown were performed in triplicate with standard deviation represented as error bars. These data are representative of three independent experiments.

Fig. S7.

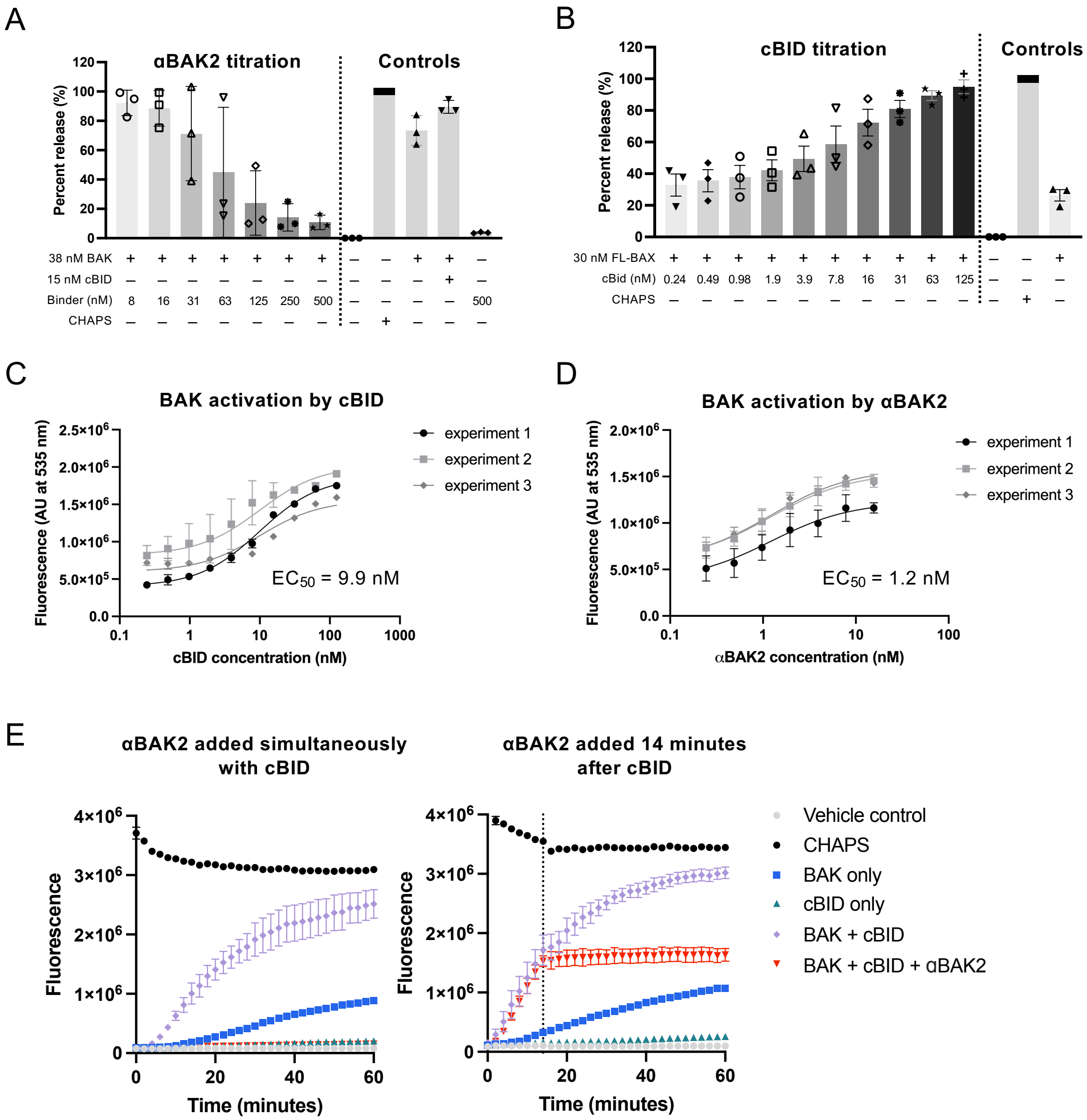

Data supporting αBAK2 inhibition and activation of BAK presented in Figure 3. (A) BAK activated on liposomes with heat treatment is inhibited by αBAK2 at concentrations comparable to BAK activated with cBID or αBAK2 alone (Figures 3A, 3B). (B) Liposome assay showing a titration of cBID activating BAK on liposomes. Comparison of EC50 values for the activation of BAK on liposomes by cBid (C) or αBAK2 (D) with mean EC50 values indicated. (E) Liposomes were treated with BAK (38 nM) and cBID (15 nM) with or without αBAK2 (250 nM) added simultaneously with cBID (left) or 14 minutes after cBID (right). (A, B) Bars represent mean values with standard error from 3 independent experiments shown with symbols. Each independent experiment was performed in technical triplicate. (C, D) Plots show three independent experiments performed in triplicate, with mean and standard deviation shown. (E) Plots are representative of three independent experiments, with error bars indicating standard deviation from triplicate measurements.

Fig. S8.

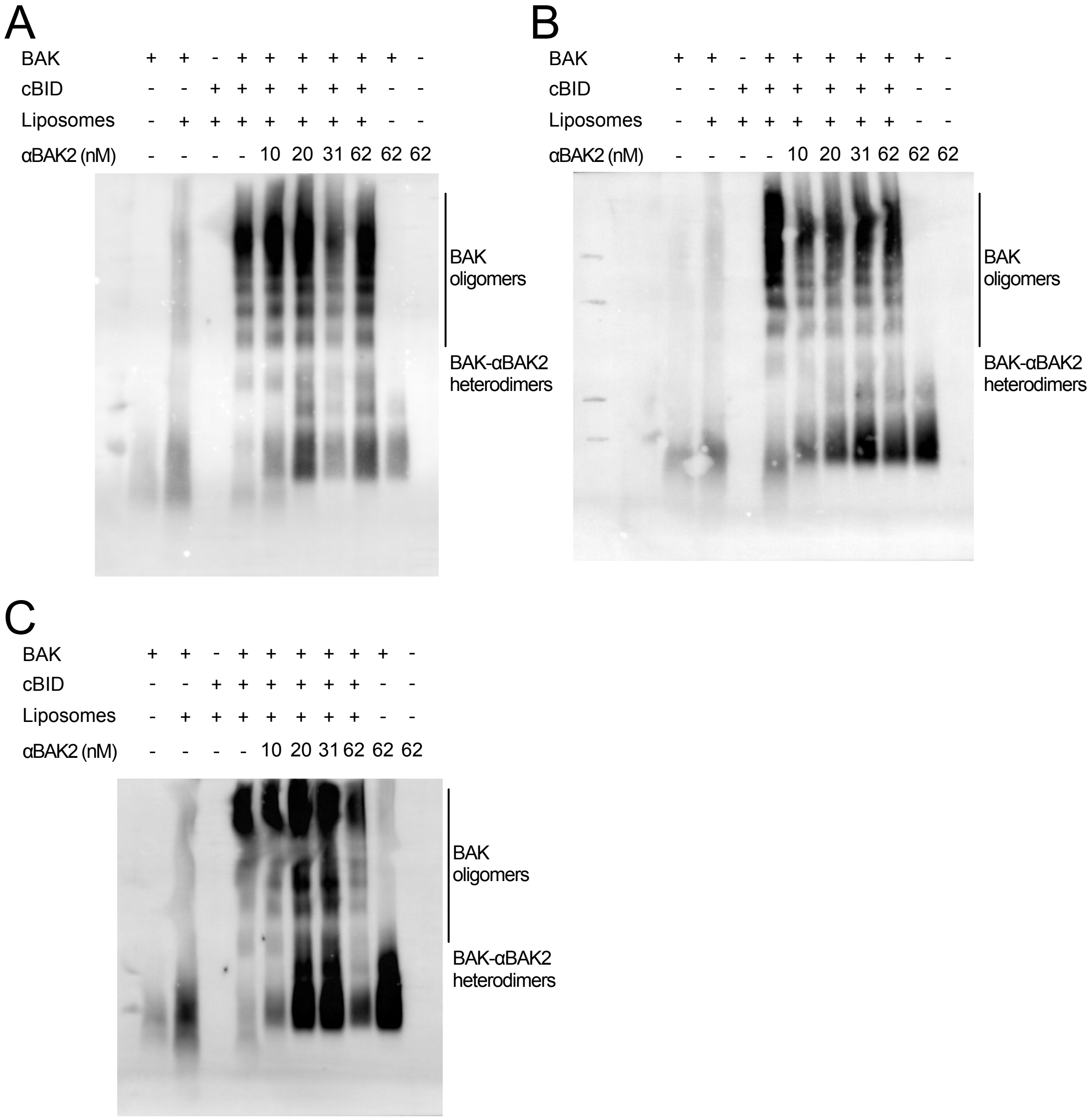

αBAK2 does not disrupt BAK oligomers that form on liposomes after activation with cBID. BAK treated with cBID for 30 minutes to form oligomers and permeabilize liposomes. After another 30 minutes various concentrations of αBAK2 were added and samples run on blue native PAGE to maintain BAK oligomers. PAGE gels were blotted for BAK showing BAK oligomer formation and some αBAK2-BAK heterodimers, but no change in intensity of the BAK oligomers.

Fig. S9.

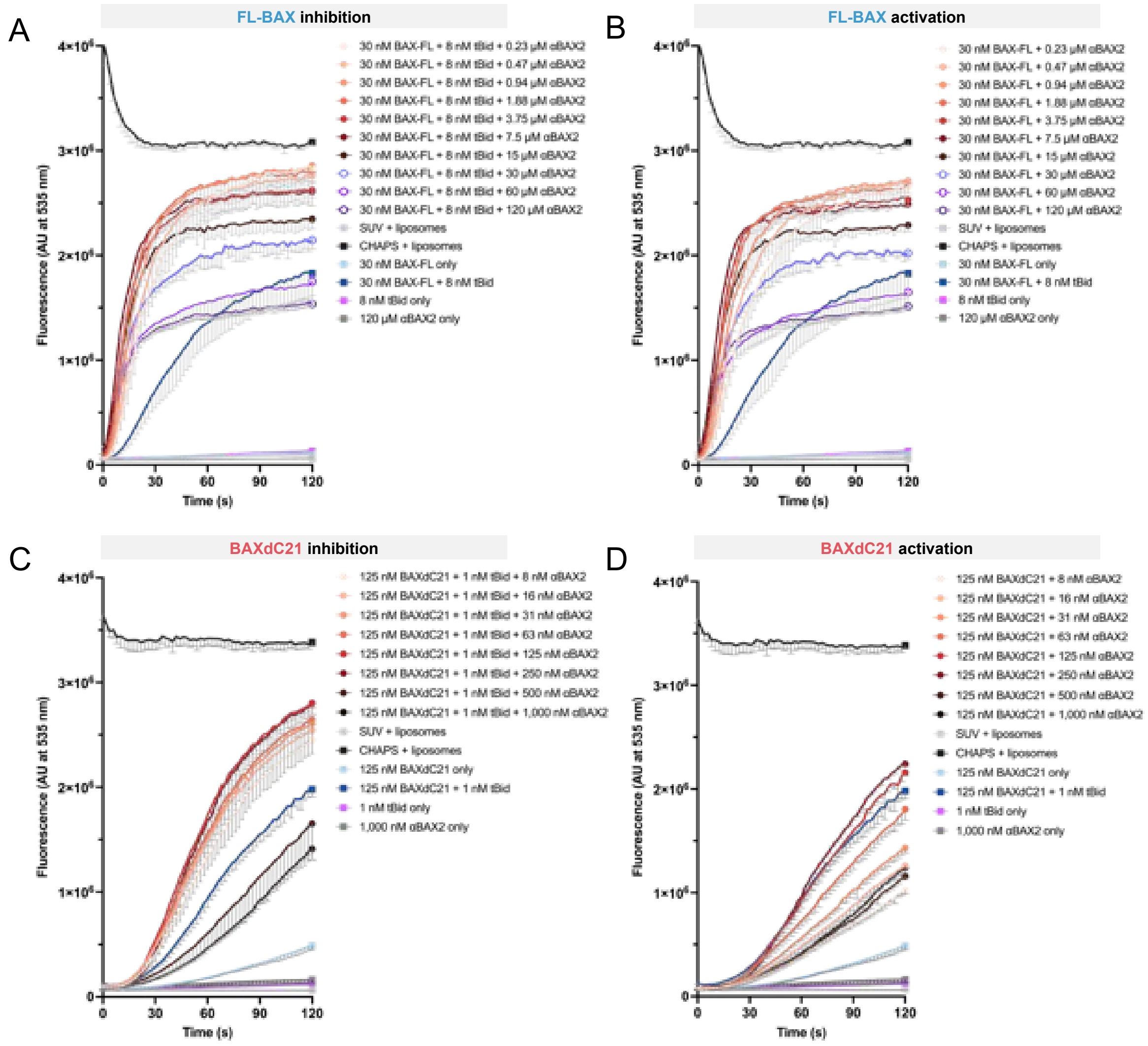

Time course showing BAX binders activate or inhibit BAX, depending on binding affinity and relative concentration. (A, C) Liposomes were treated with full length BAX (FL-BAX) or BAX lacking the C-terminal transmembrane domain (BAXdC21), an activating concentration of BOP cBID, and a titration of αBAX2 to determine its propensity to inhibit BAX-mediated permeabilization, or (B, D) treated with BAX and a titration of αBAX2 to determine its propensity to activate BAX-mediated permeabilization. Fluorescence intensity of the assay solution, as a proxy for liposome permeabilization, was monitored over time, and plotted relative to the positive control treatment (CHAPS). Data shown were performed in triplicate with standard deviation represented as error bars. These data are representative of three independent experiments.

Fig. S10.

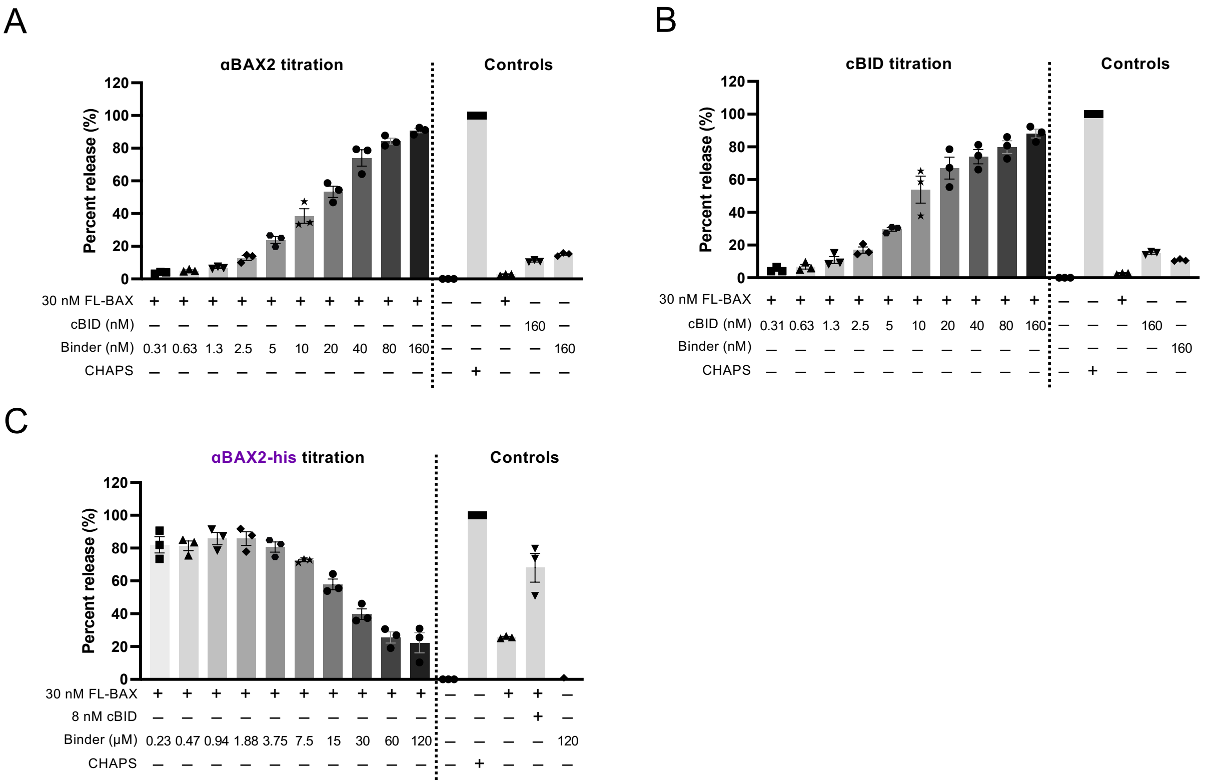

Data supporting αBAX2 activation and inhibition of BAX presented in Figure 4. FL-BAX activated on liposomes with a titration of αBAX2 (A) or cBID (B), related to Figure 4E. (C) Inhibition of FL-BAX mediated liposome release from NTA-liposomes with αBAX2-his, related to Figure 4F. Bars represent mean values with standard error from 3 independent experiments shown with symbols. Each independent experiment was performed in technical triplicate.

Fig. S11.

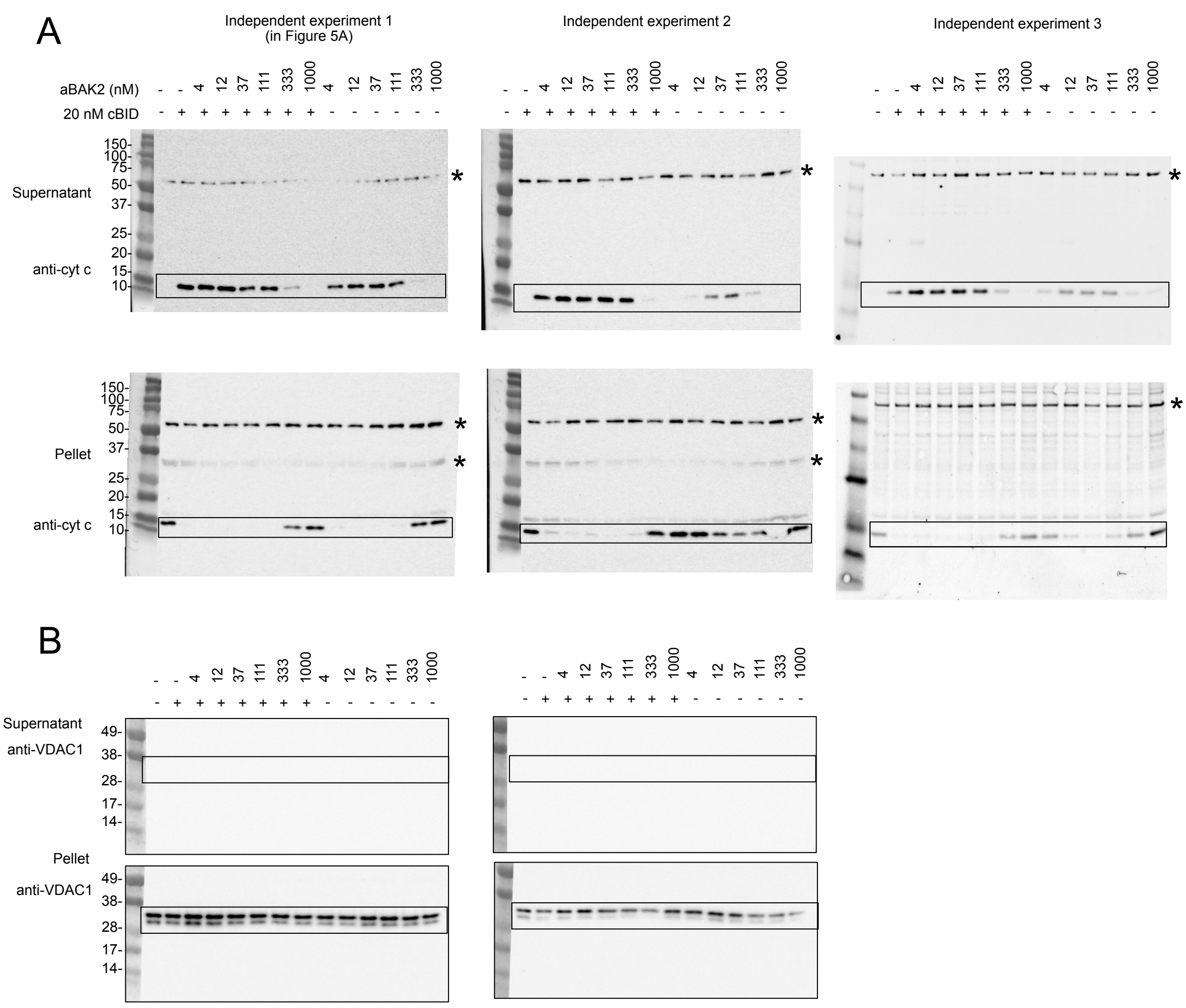

Complete blots MEF assays in Figure 5 showing αBAK2 induce or inhibit cytochrome *c* release from mitochondria, depending on concentration. The figure shows complete blots from three independent experiments of mitochondrial release assays performed in permeabilized MEF BAK/BAX double knock out cells expressing human BAK. Pellet and supernatant fraction blots were initially probed for (A) cytochrome *c* followed by (B) VDAC1. VDAC1 was used as the loading control and as a membrane protein localized to the pellet fractions. Non-specific bands in the cytochrome *c* blots are indicated (*) and molecular weight standard sizes (kDa) indicated on the left.

Fig. S12.

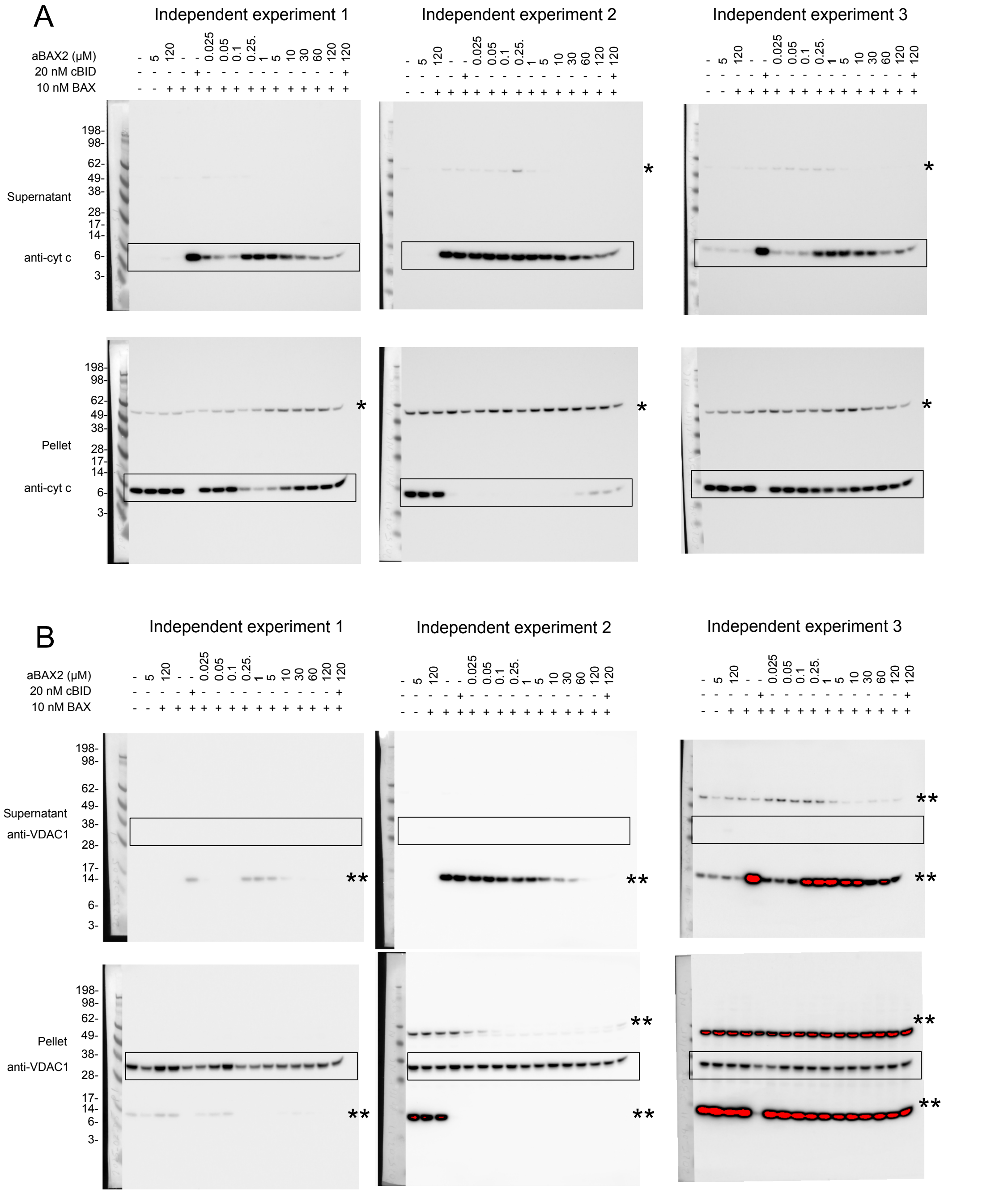

Complete blots MLM assays in Figure 5 showing αBAX2 induce or inhibit cytochrome *c* release from mitochondria, depending on concentration. The figure shows complete blots from three independent experiments of mitochondrial release assays performed on isolated mitochondria from MLM BAK knock out trated with recombinat human FL-BAX. Pellet and supernatant fraction blots were initially probed for (A) cytochrome *c* followed by (B) VDAC1. VDAC1 was used as the loading control and as a membrane protein localized to the pellet fractions. Non-specific bands in the cytochrome *c* blots are indicated (*), residual chemiluminescence from the cytochrome c blots is indicated in the VDAC1 blots (**) and molecular weight standard sizes (kDa) indicated on the left.

Table S1.

Design name	energy (REU)	energy (ddG)	complementarity	atoms	accessible	sasa	target	or variant of	Expression	YSD screen<br>Binds BAK***	Binds BAX***
BAK-CDP01	-534.8	-57.3	0.66	3	2917	-19.7	BAK	2M5B	+++	++	++
BAK-CDP02	-524	-42.5	0.56	3	2888	-14.7	BAK	2M5B	+++	++	+
BAK-CDP03	-519.7	-59.4	0.62	3	2459	-24.2	BAK	2M5B	+++	-	-
BAK-CDP04	-526.9	-53.6	0.56	4	2452	-21.9	BAK	2M5B	+++	-	-
BAK-CDP05	-547.5	-53.2	0.66	2	2471	-21.5	BAK	4OYD*	+++	-	-
BAK-CDP06	-520.7	-41.1	0.61	0	2192	-18.7	BAK	4OYD*	+++	-	-
BAK-CDP07	-526.6	-46	0.62	3	2272	-20.3	BAK	4OYD*	+++	-	-
BAK-CDP08	-532.3	-37.3	0.66	1	2286	-16.3	BAK	4OYD*	+++	-	-
BAK-CDP09	-501.2	-32.5	0.63	3	2321	-14	BAK	4OYD*	+++	-	-
BAK-CDP10	-533.2	-70.9	0.72	5	2627	-27	BAK	4OYD**	+++	-	+
BAK-CDP11	-514.3	-44.2	0.68	3	2349	-18.8	BAK	4OYD**	+++	-	+
BAK-EC1M01	-526.9	-29.2	0.64	5	2449	-11.9	BAK	BAK-CDP02			
BAK-EC1M02	-552.8	-35.5	0.59	3	2433	-14.6	BAK	BAK-CDP02			
BAK-EC1M03	-532.3	-38.6	0.64	3	1671	-23.1	BAK	BAK-CDP02			
BAK-EC1M04	-534.7	-35.8	0.58	3	2214	-16.2	BAK	BAK-CDP02			
BAK-EC1M05	-539.2	-36.4	0.57	7	2642	-13.8	BAK	BAK-CDP02			
BAK-EC1M06 (αBAK1)	-525.4	-24	0.65	3	2453	-9.8	BAK	BAK-CDP02			
BAK-EC2M01 (αBAK2)	-511.3	-26.6	0.49	4	2359	-11.3	BAK	BAK-EC1M06			
BAK-EC2M02	-505.1	-20.3	0.53	5	2416	-8.4	BAK	BAK-EC1M06			
BAK-EC2M03	-498.9	-38.5	0.5	6	2528	-15.2	BAK	BAK-EC1M06			
BAK-EC2M04	-505.8	-35.5	0.5	3	2330	-15.2	BAK	BAK-EC1M06		NA	
BAK-EC2M05	-501.4	-31.9	0.54	5	2325	-13.7	BAK	BAK-EC1M06			
BAK-EC2M06	-511.4	-39.6	0.55	5	2588	-15.3	BAK	BAK-EC1M06			
BAK-EC2M07	-492.6	-47.1	0.56	7	2562	-18.4	BAK	BAK-EC1M06			
BAK-EC2M08	-503.3	-29.5	0.54	3	2562	-11.5	BAK	BAK-EC1M06			
BAK-EC2M09	-511.7	-36.2	0.47	4	2483	-14.6	BAK	BAK-EC1M06			
BAK-EC2M10	-500.5	-30.8	0.5	3	2347	-13.1	BAK	BAK-EC1M06			
BAK-EC2M11	-503.3	-29.6	0.52	3	2349	-12.6	BAK	BAK-EC1M06			
BAK-EC2M12	-496.5	-25.9	0.48	3	2415	-10.7	BAK	BAK-EC1M06			
BAK-EC2M13	-510.5	-30	0.55	4	2335	-12.9	BAK	BAK-EC1M06			
BAX-CDP01	-475.9	-48	0.67	2	2187	-21.9	BAX	1F16	+++	-	+++
BAX-CDP02	-502.4	-61.2	0.58	0	2099	-29.2	BAX	1F16	+++	-	-
BAX-CDP03	-521.7	-66.9	0.72	5	2014	-33.2	BAX	1F16	+++	-	-
BAX-CDP04	-508	-60.3	0.65	1	2196	-27.5	BAX	1F16	+++	++	+++
BAX-CDP05	-450.9	-49.2	0.64	5	2148	-22.9	BAX	4BD2	+++	-	-
BAX-CDP06	-474.6	-67.3	0.73	3	2062	-32.6	BAX	4BD2	+	-	-
BAX-CDP07	-486	-62.3	0.68	4	2325	-26.8	BAX	4BD2	+++	-	++
BAX-CDP08	-460.9	-57	0.7	2	2182	-26.1	BAX	4BD2	++	-	+
BAX-CDP09	-466	-57.5	0.65	5	1973	-29.1	BAX	4BD2	-	NA	NA
BAX-CDP10	-451.8	-61.2	0.72	5	2092	-29.2	BAX	4BD2	+	-	-
BAX-CDP11	-497.1	-49.1	0.61	4	2297	-21.4	BAX	4BD6	++	-	++
BAX-CDP12	-476.5	-42.5	0.65	7	2131	-19.9	BAX	4BD6	+++	-	-
BAX-CDP13	-500.8	-52.9	0.66	4	2190	-24.2	BAX	4BD6	+	+	+
BAX-CDP14	-475.5	-45.8	0.7	6	2187	-21	BAX	4BD6	+++	-	-
BAX-CDP15	-497.2	-39.6	0.63	5	2415	-16.4	BAX	4BD6	++	-	++
BAX-CDP16	-493.3	-66	0.73	4	2647	-24.9	BAX	4OYD*	-	NA	NA
BAX-CDP17	-471.3	-54.5	0.59	3	2384	-22.9	BAX	4OYD*	+++	-	-
BAX-CDP18	-481.2	-46.4	0.68	2	2211	-21	BAX	4OYD*	-	NA	NA
BAX-CDP19	-478.8	-61.8	0.66	3	2248	-27.5	BAX	4OYD*	+++	-	-
BAX-CDP20	-485.8	-59.8	0.64	6	2581	-23.2	BAX	4OYD*	+++	-	-
BAX-CDP21	-478.8	-52.6	0.68	3	2668	-19.7	BAX	4OYD*	+++	-	+
BAX-CDP22	-463.8	-48.6	0.62	5	2462	-19.7	BAX	4OYD*	++	-	-
BAX-CDP23	-493.4	-67.7	0.65	7	2640	-25.6	BAX	4OYD*	+	-	-
BAX-CDP24	-484.8	-50.1	0.56	6	2259	-22.2	BAX	4OYD**	+++	-	++
BAX-CDP25	-469.4	-52.8	0.62	4	2270	-23.3	BAX	4OYD**	+++	-	-
BAX-CDP26	-472.5	-54.9	0.63	4	2321	-23.7	BAX	4OYD**	+++	-	+
BAX-CDP27	-463.4	-55.6	0.61	3	2177	-25.5	BAX	4OYD**	+++	+	-
BAX-CDP28	-456.1	-54.1	0.62	2	2068	-26.2	BAX	4OYD**	++	-	+
BAX-CDP29	-459.2	-50.5	0.6	5	2343	-21.6	BAX	4OYD**	+++	-	-
BAX-CDP30	-477.9	-61.1	0.69	3	2214	-27.6	BAX	4OYD**	+++	+	++
BAX-CDP31	-476.3	-61.1	0.66	2	2206	-27.7	BAX	4OYD**	+++	-	++
BAX-EC1M01 (αBAX1)	-507.3	-57.4	0.62	1	2557	-22.5	BAX	BAX-CDP01			
BAX-EC1M02	-472.9	-33.6	0.47	3	1952	-17.2	BAX	BAX-CDP01			
BAX-EC1M03	-488.2	-38.5	0.62	1	2350	-16.4	BAX	BAX-CDP01			
BAX-EC1M04	-487.2	-60.5	0.67	2	2380	-25.4	BAX	BAX-CDP01			
BAX-EC1M05	-495.4	-54.9	0.66	1	2294	-23.9	BAX	BAX-CDP01		NA	
BAX-EC1M06	-487.1	-55.6	0.64	2	2470	-22.5	BAX	BAX-CDP01			
BAX-EC2M01 (αBAX2)	-489	-49.5	0.61	2	2608	-19	BAX	BAX-EC1M01			
BAX-EC2M02	-498.6	-60	0.65	4	2826	-21.2	BAX	BAX-EC1M01			
BAX-EC2M03	-487.4	-54.4	0.6	2	2812	-19.4	BAX	BAX-EC1M01			
BAX-EC2M04	-495.4	-51.7	0.59	7	2587	-20	BAX	BAX-EC1M01			

* homology model, diversified backbone scaffold set

** homology model, original BINDI scaffold backbone

*** relative expression and binding were manually ranked, with a "–" value representing no expression/binding, and detectable expression/binding ranked from low (+) to high (+++).

YSD screening data and computational metrics for BAK- and BAX-targeting designs.

Table S2.

Name	Sequence
BAK-CDP01	GADPKKVLDKAKDQAENVVRKLKQKLEELYKEARKLDLTQDEKIELILKYIAAMLAAIGDILQAIMDAKLEADKLKKAGLVNSQQLDELKRRLEELREKAARKAEKYAREFAKKLKYG
BAK-CDP02	GADPKKVLDKAKDQAENVVRKLKQILEELYKEARKLDLTQDEKIELILRYIAAMLAAIGDILEAIRQAKEEADKLKKAGLVNSQQLDELKRRLEELRKEAARKARDYAEEFRNKLEYG
BAK-CDP03	GADPKKVLDKAKDQAENRVRELEQKLRELYKEARKLDLTQEQKDKLQKRYLQALREALKDIENAIRQAKQEADKLKKAGLVNSQQLDELKRRLEKLRQEAVIKAIALALAFIGDLEQG
BAK-CDP04	GADPKKVLDKAKDQAENRVRELEQKLRELYKEARKLDLTQEEKRKLKKRYEQALREAHKDIRNAIRQAKQEADKLKKAGLVNSQQLDELKRRLEKLRKEAAIKAIALALAFIGDLEQG
BAK-CDP05	GADPKKVLDKAKDQAENRVQELRQKLEELYKEARKLDLTQEERRKLEERYRAALEEARRDIENAIRQAKQEADKLKKAGLVNSRQLKKLKQLLNVLAIAANLQAIGDAIEFEYRLEYG
BAK-CDP06	GADPKKVLDKAKDQAENRVRELKQKLEELYKEARKLDLTQEQKDKLRERYREAAEQAARDIYNAIRQAKQEADKLKKAGLVNSQQLDELKRRLEKLRAEAEVKAIAYALQFIGDLEAG
BAK-CDP07	GADPKKVLDKAKDQAENRVRELKQKLEELYKEARKLDLTQEQRRKLEERYRAALEEARKDIENAIRQAKQEADKLKKAGLVNSRQLKKLKQELNRLAIAANLQAIGDAIEFEYRLEYG
BAK-CDP08	GADPATVLIAAALQAIGDVEALREILEELYKEARKLDLTQEERRKLEERYKKAEEKARRDIYEAIKRAKERADELKKAGLVNSQQLDELKRRLERLKEQASREARELAREFQQKLEYG
BAK-CDP09	GADPKKVLDKAKDQAENRVRELAQKLEELYKEARKLDLTQEERRKLEERYRAALEEALKDIDNAIRQAKQEADKLKKAGLVNSRQLKKLKQELNRLAIAAKLQAIGDAIEFEYRLEYG
BAX-CDP01	GADPKKVLDKARDQAENRVRELAQKLEELYKEARKLDLTQEERRKLEKRYKAAMEEAIKDIREAIRQAKQEADKLKKAGLVNSQQLKELKQRLNELAIAAALRAIGDAIEFRRKLQYG
BAX-CDP02	GADPAEVLIAAALRAIGDVIELREILKELYKEARKLDLTQEQRRKLEERYRAAMEKAQANIYKAIKQAKEEADKLKKAGLVNSQQLDELKKRLEKLRQEASRKARDDAREFQQKLEYG
BAX-CDP03	GADPAEVLIAAALRAIGDVIELRKILEELYKEARKLDLTQEERRKLEERYRAAMEKAQANIYNAIKQAKEEADKLKKAGLVNSQQLDELKKRLEKLRKQASKKARDDAREFQQKLEYG
BAX-CDP04	GADPKKVLDKARDQAENRVRELAQKLEELYKEARKLDLTQEERRKLEERYRAAMEEAIEDIKKAIKQAKQEADKLKKAGLVNSQQLEELKRELNILAIAAALRAIGDAYEFRRRLQYG
BAX-CDP05	GANPLEVLIAAMLAAIGDVEALRQILNELYKEARKLDLTQEQRRKLEERYEAAMRKAQEAIRQAIEAAKREADKLKKAGLVNSQQLDELKRRLEKLKENASRQARDYAREFQQKLEYG
BAX-CDP06	GANPLEVLIAAMLAAIGDVIALREILKELYKEARKLDLTQEERRKLEERYKAAREKARENIRAAIEEAKKEADKLKKAGLVNSQQLDELKRRLERLKKEASRQARDYAREFQQKLEYG
BAX-CDP07	GADPKKVLDKARKQAIEVVARLAKKLKELEKEARKLDLTQEERRKLEKRYQAAMQEARDDIKNAIRQAKQEADKLKKAGLVNSDQLRELKNRLIALAIAALLAAIGDAYEFERRLEYG
BAX-CDP08	GANPLEVLIAATLAAIGDVHALRAILQELYKEARKLDLTQEQRRKLEERYKQAREEARERIRAAIEAAKREADKLKKAGLVNSQQLDELKRRLEKLKEKASRQARDYAREFQQKLEYG
BAX-CDP09	GANPLEVLIAAMLAAIGDVIALREILRELYKEARKLDLTQEERRKLEKRYEAAMKKAQKNIEAAIREAKKEADKLKKAGLVNSQQLDELKRRLEKLKKEASRQARDYAREFQQKLEYG
BAX-CDP10	GANPLEVLIAAMLAAIGDVYALKAILDELYKEARKLDLTQEQRRKLEKRYKAAMDKARKAIRDAIKAAKEEADKLKKAGLVNSQQLDELKRRLERLKKEASRQARDYAREFQQKLEYG
BAX-CDP11	GADPKKVLDKARKQADQRVQELRKKLEELYKEARKLDLTQEQRRKLEERYRAAMEEAIKDIRNAIRQAKQEADKLKKAGLVNSQQLRRLKTLLNILAIAAALRAIGDAEAFRQKLEYG
BAX-CDP12	GANPAEVLIAAALMAIGDVEALRQELNELYKEARKLDLTQEERRKLEERYRAAMEKAQEAIKRAIKAAKEEADKLKKAGLVNSQQLDKLKRELEKLRQEASRLARDYAREFQQKLEYG
BAX-CDP13	GADPKKVLDKARKQAEQRVRELAKKLKELKKEARKLDLTQEQRRKLEERYRAAMEEAIKDIRNAIRQAKQEADKLKKAGLVNSQQLRRLKTLLNILAIAAALMAIGDAFQFEQDLEYG
BAX-CDP14	GANPAEVLIAAALMAIGDVHALREELQELYKEARKLDLTQEERRKLEERYRAAMEKAQQAIEDAIKAAKEEADKLKKAGLVNSQQLDKLKRELEKLKREASRQARDYAREFQQKLEYG
BAX-CDP15	GADPKKVLDKARKQALQRVQELRKKLEELEKEARKLDLTQEQRRKLEERYRAAMEEAIKDIENAIRQAKQEADKLKKAGLVNSQQLRRLKTLLNILAIAAALMAIGDAQEFRDRLEYG
BAX-CDP16	GADPKKVLDKAKDQAENVVRELKQRLEELYKEARKLDLTDEEKERLKIRFIAAALLAIGDIIRAIAEAKREADKLKKAGLVNSQQLDELKRRLEELKKEAQRKAAEYAEEFYRKLQYG
BAX-CDP17	GADPKKVLDKARDQAENRVQELRQKLEELYKEARKLDLTQEQRRKLEERYEAAMKEAEKDIYNAIRQAKQEADKLKKAGLVNSDQLKELKIRLAELAIAAALLAIGDAEAFRYLLEYG
BAX-CDP18	GADPKKVLDKAKDQAENRVRELKQKLEELYKEARKLDLTQEQKNKLKERARAAAEEAAKDIENAIRQAKQEADKLKKAGLVNSQQLDELKKRLEKLKIKAAEKAIAYALLFIGDLEAG
BAX-CDP19	GADPKKVLDKARDQAENRVQELRQKLEELYKEARKLDLTQEQRRKLEERYRAAMEEAERDIKNAIRQAKQEADKLKKAGLVNSDQLKELKLRLAELAIAAALLAIGDAEAFRDLLEYG
BAX-CDP20	GADPKKVLDKAKDQAENRVRELKQELEELYKEARKLDLTDEQKEQLKIRFIAAALLAIGDIIEAIDRAKQEADKLKKAGLVNSQQLDELKRRLEELEREAARKAAEYAREFYEKLQYG
BAX-CDP21	GADPKKVLDKAKDQAENVVRELKQRLEELYKEARKLDLTDEEKRRLKIRFIAAALLAIGDIEKAIHDAKREADKLKKAGLVNSQQLDELKRRLDELRKKAQKKAAEYAREFYEKLQYG
BAX-CDP22	GADPKKVLDKAKDQAENVVRELKQILEELYKEARKLDLTDEEKRQLKIRFIAAALLAIGDIIKAIYDAKAEADKLKKAGLVNSQQLDELKRRLEELERKAAEKAARLAREFYKKLQYG
BAX-CDP23	GADPKKVLDKAKDQAENRVRELKQILEELYKEARKLDLTDEEKRRLKIRFIAAALLAIGDIIVAIAEAKREADKLKKAGLVNSQQLDELKRRLEELEREAARKAAELAREFYEKLQYG
BAK-CDP10	GADPKKVLDKAKDQAENRVRELKQKLEELYKEARKLDLTEEMRKKLELRFIAAMLQAIGDIINAIYKAKQEADKLKKAGLVNSQQLDELKRRLEELANEAARKARDLAQEFQEKLQYG
BAK-CDP11	GADPKKVLDKAKDQAENRVRELKQKLEELYKEARKLDLTDEMKKKLELRFIAAMLQAIGDIINAIYKAKQEADKLKKAGLVNSQQLDELKRRLEELAKEAARKAEELAKEFQKKLQYG
BAX-CDP24	GADPKKVLDKAKDQAENRVRELKQKLEELYKEARKLDLTSDMEKKLRLRFIAALLAAIGDIAHAIYQAKQEADKLKKAGLVNSQQLDELKRRLEELKKEALRKAEDAAREFQEKLEYG
BAX-CDP25	GADPKKVLDKAKDQAENRVRELKQKLEELYKEARKLDLTEDMRKKLELRFIAALLAAIGDIAQAIYQAKQEADKLKKAGLVNSQQLDELKRRLEELKKEALRKAYEAAREFQEKLEYG
BAX-CDP26	GADPKKVLDKAKDQAENRVRELKQKLEELYKEARKLDLTEDMRKDLELRFIAALLAAIGDIAHAIYQAKQEADKLKKAGLVNSQQLDELKRRLEELRKEALRKAEDAAREFQEKLEYG
BAX-CDP27	GADPKKVLDKAKDQAENRVRELKQKLEELYKEARKLDLTERMKEELLLRFIAAALMAIGDIRAAIYQAKQEADKLKKAGLVNSQQLDELKRRLEELRKEALRKARDAAEEFQRKLEYG
BAX-CDP28	GADPKKVLDKAKDQAENRVRELKQKLEELYKEARKLDLTEEMKRELLLRFIAALLMAVGDILAAIYQAKQEADKLKKAGLVNSQQLDELKRRLEELRKEALRKARDAAREFQEKLEYG
BAX-CDP29	GADPKKVLDKAKDQAENRVRELKQKLEELYKEARKLDLTRDMEKELRLRFIAALLMAQGDIDQAIYQAKQEADKLKKAGLVNSQQLDELKRRLEELKKEARRKARDAAKEFQDKLEYG
BAX-CDP30	GADPKKVLDKAKDQAENRVRELKQILEELYKEARKLDLTDEMKKKLKLRFIAAMLLAIGDILNAIYQAKQEADKLKKAGLVNSQQLDELKRRLEELKKEAQRKASEAAREFLEKLEYG
BAX-CDP31	GADPKKVLDKAKDQAENRVRELKQILEELYKEARKLDLTEEMRKKLELRFIAAMLLAIGDILNAIYQAKQEADKLKKAGLVNSQQLDELKRRLEELKKEAQRKASEAAEEFRRKLEYG
BAK-EC1M01	GADPKKVLDKAKDQMENVVRKLKQILEELYKEARKLDLTQHEKIELILRYIVAHLAAIGDIEEAIREAKEEAQKLKKAGLVNSQQLREFKRRLEELHKEADRKRRDYAEEFRNKLEYG
BAK-EC1M02	GADPKKVLDKFKDQAENVVRKLKQILEELIKEARKLDLTQHEKIELIIRYIVAHLAAIGDIEEAIREAKEEADKLKKAGLVNSQQLDEFKRRLEELHKEADRKRRDYAEEFRNKLEYG
BAK-EC1M03	GADPKKVLDKAKDQMENVVRKLKQILEELAKEARKLDLTQHEKIELILRYIVAHLAAIGDIEEAIREAKEEADKLKKAGLVNSQQLREFKRRLEELHKEADRKRRDYAEEFRNKLEYG
BAK-EC1M04	GADPKKVLDKFKDQAENVVRKLKQILEELAKEARKLDLTQHEKIELILRYIVAHLAAIGDIEEAIREAREEADKLKKAGLVNSQQLRELKRRLEELHKEADRKRRDYAEEFRNKLEYG
BAK-EC1M05	GADPKKVLDKAKDQAENVVRKLKQILEELAKEARKLDLTQHEKIELILRYIVAHLAAIGDIEEAIREAREEAQKLKKAGLVNSQQLDEFKRRLEELHKEADRKRRDYAEEFRNKLEYG
BAK-EC1M06 (αBAK1)	GADPKKVLDKAKDQMENVVRKLKQILEELAKEARKLDLTQDEKIELILRYIVAHLAAIGDIEEAIREAKEEADKLKKAGLVNSQQLDEFKRRLEELHKEADRKRRDYAEEFRNKLEYG
BAK-EC2M01 (αBAK2)	GADPKKVLDKADDQMENVVVTLKQELEELAKERRKLDLTQSEKIELKLRYIVAHLAAIGDIEEAIREAKEEADKLKKAGLVNSQQFAEFKRRLEELHKEADRKRRDYAEEFRNKLEYG
BAK-EC2M02	GADPKKVLDQAKDQMENVVLTLKFELEELAKEARKLDLTQSEKIELKLRYIVAHLAAIGDIEEAIREAKEEADKLKKAGLVNSQQFSEFKRRLEELHKEADRKRQDYAEEFRNKLEYG
BAK-EC2M03	GADPKFVLDKADDQMENVVRTLKFELEELAKERRKLDLTQSEKIELKLRYIVAHLAAIGDIEEAIREAKEEADKLKKAGLVNSQQFAEFKRRLEELHKEADRKRQDYAEEFRNKLEYG
BAK-EC2M04	GADPKKVLDQAKDQMENVVVTLKQELEELAKERRKLDLTQSEKIELKLRYIVAHLAAIGDIEEAIREAKEEADKLKKAGLVNSQQLSEFKRRLEELHKEADRKRQDYAEEFRNKLEYG
BAK-EC2M05	GADPKKVLDQAKDQMENVVVTLKFELEELAKERRKLDLTQSAKIELKLRYIVAHLAAIGDIEEAIREAKEEADKLKRAGLVNSQQFDEFKRRLEELHKEADRKRQDYAEEFRNKLEYG
BAK-EC2M06	GADPKFVLDQAKDQMENVVVTLKQELEELAKERRKLDLTQSAKIELKLRYIVAHLAAIGDIEEAIREAKEEADKLKKAGLVNSQQLSEFKRRLEELHKEADRKRADYAEEFRNKLEYG
BAK-EC2M07	GADPKKVLDQANDQMENVVRTLKQELEELAKERRKLDLTQSAKIELKLRYIVAHLAAIGDIEEAIREAKEEADKLKKAGLVNSQQFSEFKRRLEELHKEADRKRVDYAEEFRNKLEYG
BAK-EC2M08	GADPKFVLDQAKDQMENVVLTLKQELEELAKEARKLDLTQSAKIELKLRYIVAHLAAIGDIEEAIREAKEEADKLKRAGLVNSQQFSEFKRRLEELHKEADRKRRDYAEEFRNKLEYG
BAK-EC2M09	GADPKFVLDKADDQMENVVLTLKQELEELAKEARKLDLTQSEKIELKLRYIVAHLAAIGDIEEAIREAKEEADKLKKAGLVNSQQFAEFKRRLEELHKEADRKRVDYAEEFRNKLEYG
BAK-EC2M10	GADPKKVLDQANDQMENVVRTLKFELEELAKERRKLDLTQSEKIELKLRYIVAHLAAIGDIEEAIREAKEEADKLKRAGLVNSQQFAEFKRRLEELHKEADRKRADYAEEFRNKLEYG
BAK-EC2M11	GADPKKVLDKANDQMENVVLTLKFELEELAKERRKLDLTQSEKIELKLRYIVAHLAAIGDIEEAIREAKEEADKLKKAGLVNSQQFSEFKRRLEELHKEADRKRVDYAEEFRNKLEYG
BAK-EC2M12	GADPKKVLDQAKDQMENVVVTLKQDLEELAKERRKLDLTQSEKIELKLRYIVAHLAAIGDIEEAIREAKEEADKLKKAGLVNSQQFAEFKRRLEELHKEADRKRQDYAEEFRNKLEYG
BAK-EC2M13	GADPKKVLDKAKDQMENHVLTLKQELEELAKERRKLDLTQSAKIELKLRYIVAHLAAIGDIEEAIREAKEEADKLKKAGLVNSQQFAEFKRRLEELHKEADRKRQDYAEEFRNKLEYG
BAX-EC1M01 (αBAX1)	GADPKKVLDKARDQAENRVRELAQKLEELYKEARKLDLTQEERRKLEKRYKAAMEEAIKDIHEAIEQAKEEADKLKDAGLVNEQQLKELKQRLNELAIAAAFRASGDTIEFRRKLQYG
BAX-EC1M02	GADPKKVLDKARDQAENRVRELAQKLEELYKEARKLDLTQEERRKLESRYKAEMEEAIKDIHEAIEQAKEEADKLQDAGLVNSQQLKELTQRLNELAIAAAFRASGDAIEFRRKLQYG
BAX-EC1M03	GADPKKVLDKARDQAENRVRELAQKLEELYKEARKLDLTQEERRKLEKRYKAAMEEAIKDIHEEISQAKEEADKLHKAGLVNSQQLKELKQRLNELAIAAAFRASGDAIEFRRKLQYG
BAX-EC1M04	GADPKKVLDKARDQAENRVRELAQKLEELYKEARKLDLTQEERRKLEKRSKAAMEEAIKDIREEISQAKEEADKLKDAGLVNEQQLKELTQRLNELAIAAAFRASGDTIEFRRKLQYG
BAX-EC1M05	GADPKKVLDKARDQAENRVRELAQKLEELYKEARKLDLTQEERRKLEKRYKAAMEEAIKDIHEEIHQAKQEADKLQDAGLVNSQQLKELKQRLNELAIAAAFRASGDTIEFRRKLQYG
BAX-EC1M06	GADPKKVLDKARDQAENRVRELAQKLEELYKEARKLDLTQEERRKLEKRYKAAMEEAIKDIREAISQAKEEADKLQKAGLVNEQQLKELKQRLNELAIAAAFRASGDTIEFRRKLQYG
BAX-EC2M01 (αBAX2)	GADPKKVLDKARDQAENRVRELRQKLTELYKEARKLDLTQEERRKLIKRYKAAMNEAIKDIHEAIEQAKEEADKLKDAGLVNEQQLKELKQRLNEKALAAAFRASGDTWHARRKLQYG
BAX-EC2M02	GADPKKVLDKARYQAENRVRELRQKLTELYKEARKLDLTQEERRKLSKRYKAAMNEAIKDIHEAIEQAKEEADKLKDAGLVNEQQLKELKQRLNEKALAAAFRASGDTWHFRRKLQYG
BAX-EC2M03	GADPKKVLDKARYQAENRVRELRQKLEEYYKEARKLDLTQEERRKLIKRYKAAMNEAIKDIHEAIEQAKEEADKLKDAGLVNEQQLKELKQRLNEKALAAAFRASGDTWHARRKLQYG
BAX-EC2M04	GADPKKVLDKARDQAENRVRELRQKLEEYYKEARKLDLTQEERRKLSKRYKAAMEEAIKDIHEAIEQAKEEADKLKDAGLVNEQQLKELKQRLNEKALAAAFRASGDTWHARRKLQYG

Design sequences targeting pro-apoptotic BCL2 members BAK and BAX.

Table S3.

	Bak	Bax	Bcl-2	Bcl-xL
**MBP-BimBH3**	4,000 ± 2,000	500 ± 100	0.9 ± 0.2	1.6 ± 0.1
**MBP-BidBH3**	> 4 μM	464 ± 4	47 ± 5	2.3 ± 0.4

Dissociation constants (KDs) for native interactions with the BH3-binding cleft of Bak and Bax. All values are in nanomolar. Binding affinities for Bcl-2 and Bcl-xL were determined with multiple-concentration binding titrations using BLI.

Table S4.

Library	Target	Sort	Incubation conditions		
			Target conc.	Competitor conc. (nM)	Specificity
BAK-CDP02 SSM	BAK	1 (affinity)	40	NA	NA
		1 (specificity)	100	2	0.02
		2 (affinity)	10	NA	NA
		2 (specificity)	50	4	0.08
BAK-CDP02 combinatorial	BAK	1	25	4	0.16
		2	10	16	1.6
		3	10	16	1.6
		4	5	25	5
		5	5	32	6.4
		6	2	32	16
αBAK1 SSM	BAK	1 (affinity)	1	NA	NA
		1 (specificity)	2	64 nM Bcl-2, 32 nM all others	32 for Bcl-2, 16 for all others
		2 (affinity)	0.5	NA	NA
		2 (specificity)	1	80 nM Bcl-2, 40 nM all others	80 for Bcl-2, 40 for all others
αBAK1 combinatorial	BAK	1	1	80 nM Bcl-2, 40 nM all others	80 for Bcl-2, 40 for all others
		2	1	120 nM Bcl-2, 40 nM all others	120 for Bcl-2, 40 for all others
		3	1	240 nM Bcl-2, 40 nM all others	240 for Bcl-2, 40 for all others
		4	1	240 nM Bcl-2, 40 nM all others	240 for Bcl-2, 40 for all others
		5	1	240 nM Bcl-2, 40 nM all others	240 for Bcl-2, 40 for all others
BAX-CDP01 SSM	BAX	1 (affinity)	10	NA	NA
		1 (specificity)	25	8	0.32
		2 (affinity)	4	NA	NA
		2 (specificity)	16	16	1
BAX-CDP01 combinatorial	BAX	1	16	8	0.5
		2	8	25	3.125
		3	4	25	6.25
		4	2	50	25
		5	1	50	50
		6	1	50	50
αBAX1 SSM	BAX	1 (affinity)	5	NA	NA
		1 (specificity)	6	100 nM Bfl-1, 50 nM all others	16.7 Bfl-1, 8.3 all others
		2 (affinity)	0.5	NA	NA
		2 (specificity)	1	80 nM Bfl-1, 40 nM all others	80 for Bfl-1, 40 for all others
αBAX1 combinatorial	BAX	1	1	120 nM Bfl-1, 40 nM all others	120 Bfl-1, 40 all others
		2	0.5	160 nM Bfl-1, 40 nM all others	320 Bfl-1, 80 all others
		3	0.5	280 nM Bfl-1, 40 nM all others	560 Bfl-1, 80 all others
		4	0.5	280 nM Bfl-1, 40 nM all others	560 Bfl-1, 80 all others
		5	0.5	280 nM Bfl-1, 40 nM all others	560 Bfl-1, 80 all others

Sort conditions for BAK- and BAX-targeting in vitro evolution experiments.

Table S5.

Library	Target	Theoretical Diversity	Mutations included in library	Mutations in best selected variant
BAK-CDP02 SSM	BAK	2,321	All single mutants	NA
BAK-CDP02	BAK	35,831,808	All possible combinations of:	**αBAK1: A14M, Y29A, A51V,**
αBAK1 SSM	BAK	2,321	All single mutants	NA
αBAK1 combinatorial	BAK	13,934,592	All possible combinations of:	**αBAK2: K9Q, K11N, K20T, Q23F,**
BAX-CDP01 SSM	BAX	2,321	All single mutants	NA
BAX-CDP01	BAX	11,943,936	All possible combinations of:	**αBAX1: R61H, R65E, Q69E,**
αBAX1 SSM	BAX	2,321	All single mutants	NA
αBAX1 combinatorial	BAX	65,536	All possible combinations of:	**αBAX2: D12Y, A22R, L28Y, E46I,**

Mutation summary of evolved BAK- and BAX-targeting variants.

Table S6.

	BAK:aBAK2	Unbound aBAK2
**PDBID**	9CLB	8EJA
**Wavelength**	0.9537	0.97901
**Resolution range**	40.56 - 2.86 (2.96 - 2.86)*	47.71 - 2.81 (2.91 - 2.81)
**Space group**	P 1	P 41 21 2
**Unit cell**	48.50, 74.49, 76.32, 113.13, 90.15, 105.51	53.82, 53.82, 206.03, 90, 90, 90
**Total reflections**	64534 (5373)	71378 (1918)
**Unique reflections**	21041 (1921)	8020 (767)
**Multiplicity**	3.1 (2.8)	8.9 (8.4)
**Completeness (%)**	96.92 (88.31)	99.84 (99.74)
**Mean I/sigma(I)**	4.70 (1.44)	13.4 (2.5)
**Wilson B-factor**	50.7	65.95
**R-meas**	0.2008 (1.224)	0.100 (0.800)
**R-pim**	0.1101 (0.6928)	0.051 (0.411)
**CC1/2**	0.983 (0.49)	0.999 (0.907)
**Reflections used in refinement**	20975 (1874)	8010 (765)
**Reflections used for R-free**	1078 (90)	776 (74)
**R-work**	0.2212 (0.2916)	0.2590 (0.3389)
**R-free**	0.2696 (0.3583)	0.3080 (0.3557)
**Number of non-hydrogen atoms**	6403	1881
**macromolecules**	6403	1868
**Protein residues**	778	225
**RMS(bonds)**	0.004	0.002
**RMS(angles)**	0.58	0.46
**Ramachandran favored (%)**	99.87	99.1
**Ramachandran allowed (%)**	0.13	0.9
**Ramachandran outliers (%)**	0	0
**Rotamer outliers (%)**	1.52	1.02
**Clashscore**	3.76	1.85
**Average B-factor**	56.05	75.6
**macromolecules**	56.05	75.65
**Number of TLS groups**	18	1

Crystallographic data collection and refinement statistics. (*) Statistics for the highest resolution shell.
